# Whole genome sequencing and characterization of *Corynebacterium* isolated from the healthy and dry eye ocular surface

**DOI:** 10.1186/s12866-024-03517-9

**Published:** 2024-09-28

**Authors:** Maria Naqvi, Tor P. Utheim, Colin Charnock

**Affiliations:** 1 Department of Life Sciences and Health, Faculty of Health Sciences, Oslo Metropolitan University, Postbox 4, St. Olavs Plass, Oslo, 0130 Norway; 2 Department of Medical Biochemistry, Oslo University Hospital, Oslo, Norway; 3 Department of Ophthalmology, Oslo University Hospital, Oslo, Norway; 4 The Norwegian Dry Eye Clinic, Ole Vigs gate 32 E, Oslo, 0366 Norway

**Keywords:** *Corynebacterium*, Whole genome sequencing, Phenotypic characterization, Ocular isolates, Dry eye disease

## Abstract

**Background:**

The purpose of this study was to characterize *Corynebacterium* isolated from the ocular surface of dry eye disease patients and healthy controls. We aimed to investigate the pathogenic potential of these isolates in relation to ocular surface health. To this end, we performed whole genome sequencing in combination with biochemical, enzymatic, and antibiotic susceptibility tests. In addition, we employed deferred growth inhibition assays to examine how *Corynebacterium* isolates may impact the growth of potentially competing microorganisms including the ocular pathogens *Pseudomonas aeruginosa* and *Staphylococcus aureus*, as well as other *Corynebacterium* present on the eye.

**Results:**

The 23 isolates were found to belong to 8 different species of *Corynebacterium* with genomes ranging from 2.12 mega base pairs in a novel *Corynebacterium* sp. to 2.65 mega base pairs in *C. bovis.* Whole genome sequencing revealed the presence of a range of antimicrobial targets present in all isolates. Pangenome analysis showed the presence of 516 core genes and that the pangenome is open. Phenotypic characterization showed variously urease, lipase, mucinase, protease and DNase activity in some isolates. Attention was particularly drawn to a potentially new or novel *Corynebacterium* species which had the smallest genome, and which produced a range of hydrolytic enzymes. Strikingly the isolate inhibited in vitro the growth of a range of possible pathogenic bacteria as well as other *Corynebacterium* isolates. The majority of *Corynebacterium* species included in this study did not seem to possess canonical pathogenic activity.

**Conclusions:**

This study is the first reported genomic and biochemical characterization of ocular *Corynebacterium.* A number of potential virulence factors were identified which may have direct relevance for ocular health and contribute to the finding of our previous report on the ocular microbiome, where it was shown that DNA libraries were often dominated by members of this genus. Particularly interesting in this regard was the observation that some *Corynebacterium*, particularly new or novel *Corynebacterium* sp. can inhibit the growth of other ocular *Corynebacterium* as well as known pathogens of the eye.

**Supplementary Information:**

The online version contains supplementary material available at 10.1186/s12866-024-03517-9.

## Background

*Corynebacterium* is a large, heterogeneous genus of gram-positive bacteria with high GC content. Members of the genus have previously been isolated from mainly humans and animals, but also environmental samples [1, 2]. The genus *Corynebacterium* currently contains 173 valid species according to the List of Prokaryotic names with Standing in Nomenclature ( [3] accessed 29.08.2024). The most known and studied *Corynebacterium* species is *Corynebacterium diphtheriae* which is the causative agent of diphtheria [4, 5]. Other species in the genus have been recognized as opportunistic pathogens or nosocomial pathogens, and can pose a significant threat for immunocompromised patients as they have been found to be resistant to multiple drugs [2].

In humans, *Corynebacterium* have been isolated from the skin and mucosal membranes including the ocular surface, where particularly *Corynebacterium macginleyi* has been reported to cause keratitis [6]. Studies on the ocular microbiome in healthy individuals often report the presence of *Corynebacterium* [7], and in many of these studies it has been shown to be one of the most abundant genera, suggesting the genus to be a part of the normal core microbiome [8, 9]. Previous studies suggest that some *Corynebacterium* such as the putative commensal *Corynebacterium mastitidis* contribute to protection of the ocular surface by stimulating the local immune response [10]. It has also been reported that *Corynebacterium accolens* and *Corynebacterium pseudodiphtheriticum* can inhibit growth of pathogens in other bodily microbiomes [11].

Recent papers have reported that *C. accolens* and *C. pseudodiphtheriticum* [12–14] and their concentrated culture supernatants have antagonistic effects on a number of bacterial pathogens. This antagonism has been suggested to represent a barrier to infection and colonization of the nasal tract, and might, it was suggested, even be exploited in probiotic strategies. Bomar et al., [12] showed that extracellular triacylglycerol (TAG) lipase activity produces in vitro oleic acid from the TAG triolein, and that this inhibits growth of the pathogen *Streptococcus pneumoniae*. However, other as yet unidentified and proteinaceous factors are probably also involved in *Corynebacterium* antibacterial activity: Hardy et al., [11] and Menberu et al., [13] found that the culture supernatants of respectively *C. pseudodiphtheriticum* and *C. accolens* had inhibitory effects on pathogens and that this inhibition could be abolished by proteinase K treatment.

We have previously investigated the ocular microbiome in dry eye disease (DED) patients and healthy individuals [15]. We found that *Corynebacterium* was one of the most abundant genera present on the ocular surface with an average relative abundance of 31%, median relative abundance of 18.2% and a maximum relative abundance of 99.60%. A particularly startling find was that in about 12% of cases, single-species *Corynebacterium* DNA dominated (> 75%) the total ocular sequence-read library. This provides further support for the notion that *Corynebacterium* can possibly shape its surrounding microbiota - also on the ocular surface. Additionally, our logistic regression analysis suggested that *C. accolens* may be a biomarker for DED. Given the wide range of characteristics of this genus [16], we hypothesise that different species may potentially have both characteristic effects on ocular surface health and on the structure of the ocular microbiome. In an attempt to further elucidate the role(s) *Corynebacterium* plays in the ocular microbiome in health and disease, we have used whole genome sequencing (WGS) to examine the genomic characteristics of various *Corynebacterium* species isolated from DED patients and from healthy individuals [15]. We also examined a range of phenotypic traits of potential relevance for ocular health and disease including biochemical capabilities, antibiotic resistance characteristics, potential virulence factors (hydrolytic enzymes) and growth inhibiting activity both within and beyond the genus.

## Materials and methods

### Collection of isolates

Isolates were collected from DED patients recruited form the Norwegian dry eye clinic and healthy individuals from Oslo Metropolitan University as a part of a larger study [15]. Twenty-three *Corynebacterium* isolates representing 8 species from supplementary Table 1 in our previous study [15] were chosen for further in-depth characterization in the present study.

### Inoculation procedure for enzymatic tests (DNase, lipase, mucinase and protease)

*Corynebacterium* were routinely grown for analysis on brain heart infusion agar (BHIA) amended with 0.8% tween-80 (Tw-80) or on Columbia sheep blood agar (ThermoFisher). Plates were incubated at 37 ± 1 °C in a CO_2_−enriched (5%) incubator. The basic inoculation procedure was the same in all enzymatic tests: a small amount of colony material (1 µl loopful) was spread in a circular fashion in the middle of the plate (~ 3 mm diameter) and the loop was then stuck into the agar to inoculate slightly below the surface. Thereafter plates were incubated at 37 ± 1 °C in CO_2_ (5%) incubator. Production of agar plates and interpretation of enzymatic tests are described in the supporting materials and methods (2.1–2.4).

### Antibiotic susceptibility testing

Disc diffusion susceptibility testing was performed, read (zone diameter measured) and interpreted in accordance with the latest (2024) EUCAST document (https://www.eucast.org/clinical_breakpoints). In brief, strains were grown for 48 h on Mueller-Hinton agar + 5% defibrinated horse blood and 20 mg/L β-NAD (ThermoFisher). Growth was suspended in 0.9% NaCl to a density corresponding to a McFarland 0.5 (measured spectrophotometrically) and this suspension was used to inoculate the same growth medium using a sterile cotton swab as described in the EUCAST document. After placing antibiotic-containing discs on the agar (see below), plates were incubated (5% CO_2_, 35 ± 1 °C). In most cases there was insufficient growth after 16–20 h incubation to interpret the result and plates were read after a total of 40–44 h incubation. Results are reported as S (susceptible), I (intermediately susceptible) or R (clinically resistant) as specified in the guidelines (table v. 14.0 for *Corynebacterium* sp. other than *C. diphtheriae* and *C. ulcerans*). Tests were performed at least twice on separate occasions. Antibiotics tested (symbol, amount - µg) were: Clindamycin (DA, 2), Tetracycline (TE, 30), Moxifloxacin (MXF, 5), Ciprofloxacin (CIP, 5), Vancomycin (VA, 5), Penicillin (P, 1 unit), Linezolid (LZD, 10), and Rifampicin (RD, 5). Choice of antibiotics for testing was guided by the EUCAST document specifications for *Corynebacterium* spp.

### RapID™ CB PLUS system (Remel, ThermoScientific)

Biochemical profiles for the *Corynebacterium* isolates were obtained using the RapID™ CB PLUS System for *Corynebacterium* and other gram-positive coryneform bacilli. The test was performed as described in the product protocol. Plates were scored for colour changes after exactly 6 h.

### Deferred growth inhibition assay

The assay refers to a technique whereby it can be assessed if one bacterium is able to inhibit the growth of another through the production of antimicrobial compounds or through competition for nutrients [17]. In brief, 14 cm plates (BHIA + 0.8% w/v Tw-80) were centrally inoculated with a test strain (area about 1 cm) and incubated as for enzyme assays. After 5 days to allow time for copious growth and release of antimicrobials into the growth medium, the indicator strain was sprayed onto the entirety of the plate. After inoculation, plates were inverted and examined over 24–72 h for growth inhibition of the indicator organism around the test isolate. A positive result was recorded where there was obvious inhibition of the indicator organism judged visually. Test isolates (inhibitors) were selected *Corynebacterium* ocular isolates, and the indicator bacteria (inhibited) included *Corynebacterium* as well as *S. aureus* and *E. faecalis* ocular isolates [15], and reference strains (*S. aureus* DSM2569 and *P. aeruginosa* DSM22644) deposited in the German Collection of Microorganisms DSMZ (https://www.dsmz.de/). As the test and indicator organisms represented a wide range of species with great inter-species variation in the growth density, no systematic attempt was made to grade the growth inhibition. Additional details are given in the supplementary materials (2.5).

### Plasmid isolation

Plasmid isolation was performed using the High Pure Plasmid Isolation Kit (Roche, Mannheim, Germany) with slight modifications for gram-positive bacteria: *Corynebacterium* cellular material from agar plates was homogenized in the specified volume of the kit suspension buffer previously amended with lysozyme (Merck, L6876) at 1.0 mg/ml. The mix was incubated at 37 ± 1 °C for 1 h to allow cell wall digestion before proceeding to step 2 (addition of lysis buffer) in the protocol. Plasmid detection was performed in 0.75% agarose gels stained with GelGreen^®^ (Cat. No. SCT125 Merck Millipore, Darmstadt, Germany).

### Whole genome sequencing and analysis

Genomic DNA was extracted from isolates grown on BHIA amended with 0.8% Tw-80 using the GenElute bacterial Genomic DNA kit (NA2120, Sigma-Aldrich) according to the manufacturer’s instructions. The protocol for gram-positive bacteria including additional digestion with lysozyme was followed. DNA concentration and purity was measured using Qubit (Thermo Scientific). DNA libraries (150 bp paired-end) were prepared with a proprietary, modified version of NEBNext Ultra DNA prep protocol. Libraries were sequenced using Illumina sequencing (NovaSeq6000, PE150 mode) at a commercial laboratory (Eurofins Genomics, Germany). This included DNA fragmentation, end-repair and dA-tailing, adapter ligation, size selection and library amplification.

### Bioinformatics

All genomes were assembled de novo. Fastp [18] was used for quality filtering, adaptor trimming and removing short reads (< 30 bp). For quality filtering, the sliding window approach was used removing bases with an average phred score < 20. Prior to de novo assembly with SPAdes [19], reads were error-corrected and normalised based on kmer counts using bbnorm [20]. QUAST was used for quality evaluation to assess the assembly [21].

### Genomic analyses

Genome annotation was performed using the comprehensive genome analysis service by Bacterial and Viral Bioinformatics Resource Centre (BV-BRC) [22]. Assembled contigs were uploaded to the platform. The genomes were annotated using RAST tool kit (RASTtk) [23] with the PATRIC database [24] identifying proteins by gene ontology assignments [25], enzyme commission numbers [26], and by mapping to KEGG pathways [27]. BV-BRC also identifies so-called *speciality genes* such as virulence factors, antimicrobial resistance genes, and drug targets using different databases. These were as follows: CARD, NDARO, PATRIC, DrugBank, TTD, VFDB and Victors.

ResFinder 4.3.3 from Center for Genomic Epidemiology was used to detect acquired resistance genes, with a threshold of identity of 90% and a minimum length of 60% [28].

Pan and core genome analysis was performed as described previously [29]. In brief, the BPGA pipeline [30] was used for the pan and core genome analysis using the default setting with UCLUST to cluster orthologous genes (COG) and perform KEGG assignment. MUSCLE was used to perform the alignment of core genes. Graphs were produced using R.

### Phylogenomic Analysis

The Type Strain Genome Server (TYGS) (https://tygs.dsmz.de) was used to construct phylogenetic trees [31, 32]. The tool determines the intergenomic relatedness between the query genomes and genomes present in the database using the MASH algorithm [33]. Ten type strains with the lowest MASH distance per query genome are chosen. Phylogenetic trees were also constructed based on the 16S rDNA genes in the query genomes and sequences present in the database as follows: RNAmmer [34] was used to extract the 16S rDNA gene from the query genomes and BLASTed [35] against the type strains available in the TYGS database (21541 strains 30.08.2024). The bitscores were used to find the best 50 matching type strains for each query genome and used to calculate the precise distances using the Genome BLAST Distance Phylogeny approach (GBDP) under the coverage algorithm and formula d5 [36]. The 10 closest type strain genomes were identified based on these distances.

 Pairwise comparisons were performed with GBDP and intergenomic distances were inferred using the trimming algorithm and distance formula d5. One hundred replicates were calculated each. In addition, digital DNA: DNA hybridization (dDDH) values were calculated with the Genome to Genome Distance Calculator (GGDC) using the recommended settings [32, 36]. FASTME with SPR post processing was used to infer a balanced minimum evolution tree with branch support from 100 pseudo-bootstrap replicates each using the intergenomic distances [37]. The trees were rooted at the midpoint [38] and visualized with PhyD3 [39]. The figures were modified in Inkscape (https://inkscape.org/). Accession numbers for the genomes included in the trees are given in supplementary materials and methods (Sect. 2.6).

Average nucleotide identity (ANI) was calculated using JSpeciesWS online web server [40].

### Lipase and lipase-like genes present in functional genome annotations

Structurally-annotated genomes of several isolates showing lipase activity on olive oil and/or inhibition of indicator strains in the deferred growth assay, were analysed for the presence of genetic determinants of lipolytic enzymes as follows: firstly, putative lipase/esterase genes suggested in the structural annotations were recorded. However, as many sequences were annotated as hypothetical proteins, we widened our search using two strategies: (i) detection of lipase/esterase sequence motifs using the motif search tool available at GenomeNet ( https://www.genome.jp/), and InterPro (https://www.ebi.ac.uk/interpro/about/interpro/) which provide functional analysis of protein sequences by classifying them into families and predicting the presence of domains and important sites. (ii) manual, side-by-side comparisons with sequences of proposed *Corynebacterium* lipases/esterases deposited in GenBank (https://www.ncbi.nlm.nih.gov/genbank/). All sequences of interest were then further analysed using both BlastP [41] and EMBL-EBI Job Dispatcher sequence analysis tools [42] searches. A similar approach was used for the detection of putative bacteriocins or their biochemical production pathway in the genomes. Whole genomes were searched using antiSMASH (https://antismash.secondarymetabolites.org/#!/start) which enables genome-wide identification, annotation and analysis of secondary metabolite biosynthesis gene clusters.

### Prediction of secretory signal peptides

Lipase and lipase-like genes were investigated for the likelihood of bacterial signal peptides using the SignalP 6.0 server (https://services.healthtech.dtu.dk/services/SignalP-6.0/) which predicts the presence of signal peptides and the location of their cleavage sites. Predicted signal peptide sequences were removed before generation of phylogenetic trees.

### Phylogenetic tree of lipolytic enzymes

We chose for inclusion in a phylogenetic tree what we considered to be the strongest candidates for hydrolysis of olive oil (a TAG including mainly esterified oleic acid) in the lipase test (supplementary materials 2.2), and the ester bond in Tw-80 (Polyethylene glycol sorbitan monooleate) used in the growth deferred assay. Additional lipase sequences were obtained from culture collection strain genomes, usually species type strains, annotated by the NCBI prokaryotic genome annotation pipeline. The annotations are available online at the NCBI together with the genome data (accession numbers are given in supplementary Table 1). We were particularly interested in the lipases of *C. accolens* and *Corynebacterium* sp. PCR 32. Owing to its possible status as a new or novel species, we included the lipases of the closest neighbour type strains of the *Corynebacterium* sp. PCR 32, based on both 16S rDNA sequencing and whole genome comparisons using the TYGS analysis (see section phylogenomic analyses). The phylogenetic tree of the lipase sequences was generated using version 2.20 of W-IQ-Tree [43] which is an online (http://iqtree.cibiv.univie.ac.at/) phylogenetic tool for maximum likelihood (ML) analysis [44]. The initial steps of the analysis, sequence alignment and trimming, were performed using tools available at NGphylogeny.fr (https://ngphylogeny.fr ) Muscle [45] was used for multiple alignments followed by implementation of trimAI [46] for alignment curation. Using IQ-tree, ML phylogenetic trees were inferred based on the best-fit substitution model using the pipeline’s ModelFinder [47]. We estimated models of substitution using the ‘Auto’ function with FreeRate heterogeneity. Branch support was assessed using Ultrafast boostrap approximation [48, 49] with 1000 replicates and the SH-like approximate likelihood ratio test [50]. The tree directory file was uploaded to, visualized and annotated using the online version of the Interactive tree of life, iTOL [51] available at https://itol.embl.de/. The tree is not rooted, but the outgroup sequence DSM44385 (c) is drawn at the root.

## Results

### Isolation and characterization of ocular Corynebacterium

A number (23 isolates representing 8 species) of ocular *Corynebacterium* isolated from the healthy and dry eye in a previous comparison of microbiomes (see supplementary Table 1 in [15]) were chosen for in-depth polyphasic characterization. The impetus for this was that although *Corynebacterium* is often considered a major component of ocular microbiomes, we found significant ocular dysbioses where *Corynebacterium* came to dominate ocular DNA libraries [15]. We show below the presence of enzyme activities potentially relevant to invasion and colonization and which may represent virulence factors, as well as clinically important antibiotic resistance features. We found also that several isolates, particularly *Corynebacterium* sp. PCR 32, were able to inhibit other ocular bacteria in vitro.

### Enzyme activities

 The presence of a number of extracellular enzyme activities (such as lipase, DNase, protease and mucinase) which are known to contribute to the processes of host invasion and colonization in a wide range of microbial species were investigated. Table 1 summarises the results of the agar-based tests for detection of enzyme activities.

Salient findings were that *C. accolens* isolates were found to be strongly lipolytic (Table 1). The major lipase associated with this species activity is probably also involved in outcompeting other ocular isolates (see deferred growth assay). Underlying genetic determinants of lipolytic activity were found in the genomes and these are singled out for attention below.

The *C. propinquum* isolates were the only isolates where DNase activity was detected (Table 1). The agar-based assay showed weak mucinase activity in *C. accolens* and *Corynebacterium* sp. PCR 32. Only the latter isolate showed proteinase activity on skimmed milk proteins.

**Table 1:** Overview of enzyme activities of *Corynebacterium* isolates

Strain	Isolate origin*	Identification based on WGS	Protease^a^	Lipase^b^	DNase^c^	Mucinase^d^
PCR1	P20	*C. propinquum*	-	+	(+)	-
PCR 8	P20	*C. propinquum*	-	+	(+)	-
PCR 11	P20	*C. propinquum*	-	+	+	-
PCR 2	P51	*C. marquesiae*	-	-	-	-
PCR 3	C9	*C. marquesiae*	-	-	-	-
PCR25	P51	*C. marquesiae*	-	-	-	-
PCR26	C4	*C. marquesiae*	-	-	-	-
PCR27	C9	*C. marquesiae*	-	-	-	-
PCR4	P48	*C. accolens*	-	+	-	+
PCR19	P48	*C. accolens*	-	+	-	+
PCR20	P48	*C. accolens*	-	+	-	+
PCR22	P10	*C. accolens*	-	+	-	(+)
PCR23	P10	*C. accolens*	-	+	-	+
PCR31	P48	*C. accolens*	-	+	-	+
PCR7	P33	*C. macginleyi*	-	(+)	-	-
PCR6	P30	*C. sanguinis*	-	-	-	-
PCR14	C4	*C. mastitidis*	-	-	-	-
PCRF	C4	*C. mastitidis*	-	-	-	-
PCR21	C4	*C. mastitidis*	-	-	-	-
PCR32	P19	*Corynebacterium* sp.	+	(+)	-	(+)
PCR37	P25	*C. bovis*	-	-	-	-
PCR38	P46	*C. bovis*	-	-	-	-
PCRi	P2	*C. bovis*	-	-	-	-
PCR39	P25	*C. bovis* (identification based on 16 S rDNA and *rpob* sequencing)	-	-	-	-

^a^*Corynebacterium *sp. PCR 32 produced zones of complete clearing after 72h of incubation. No other isolate produced clearing or partial clearing in the agar

^b^A variety of effects were obtained in the agar making a definitive interpretation of lipase production somewhat difficult. Isolates identified as *C. propinquum *ultimately produced a ring of complete clearing and weakly fluorescent orange haloes. Isolates classified as *C. accolens *produced strongly fluorescent orange haloes. The single isolates of *C. macginleyi *and *Corynebacterium *sp. (PCR 32) ultimately produced thin, reddish glowing haloes, and are tentatively scored as + for lipase production. All other isolates gave no effects in the agar and were easily scored as negative for lipase production

^c^Zones (weak) of clearing were seen only with isolates identified as *C. propinquum*

^d.^No *Corynebacterium *isolates produced zones of complete clearing in the mucin-containing agars. However, several gave zones which resembled partial clearing on mucin-brain heart infusion agar. These are scored as positive in the table on this basis. However, in the case of *Corynebacterium *sp. (PCR 32) a weak zone of partial clearing was most pronounced on R_2_A-mucin agar. Subsequent contrast staining with CaCl_2 _did not reveal complete clearing for any of the isolates. Only the control strain *P. aeruginosa* ATCC 15692 showed a zone (narrow) of complete clearing after CaCl_2_treatment. *P indicates patient sample, C indicates control sample. Numbers (P20 etc…) and patient/control characteristics (dry eye severity) are detailed in our previous study [15]

### Antibiotic susceptibility tests

Supplementary Table 2 summarises the results of disc diffusion susceptibility tests (supplementary results 3.1). All isolates were suseptible to Tetracycline (TE30), Moxifloxacin (MXF5), Vancomycin (VA5) and linzeolid (LZD10). All isolates were also found to be intermediately suseptible to Ciprofloxacin (CIP5). The majority of isolates were resistant to Clindamycin (DA2) except for 2 of the *C. accolens* isolates (PCR 22 and 23), the *C. macginleyi* isolate (PCR 7), *C. sanguinis* isolate, *Corynebacterium* sp. (PCR 32), and *C. bovis.* All isolates except PCR 3 and PCR 27 (*C. marquesiae*) were suseptible to Rifampicin (RD5). Resistance to rifampicin has been associated with substitutions in the rifampicin binding site of the rpoB protein. This possibility is examined further in supplementary results 3.2.

### Biochemical profiles of Corynebacterium isolates

Differences and similarities between 23 ocular *Corynebacterium* isolates with respect to enzymatic and metabolic properties are shown in supplementary Table 3. The table summarizes the results of biochemical profiling of *Corynebacterium* ocular isolates using the RapID™ CB Plus kit. In some cases it was considered that a definitive interpretation (i.e., +, - for colour development) could not be made and these results are scored as +/-. The recommended control strain ATCC 10701 gave the expected results for all tests. Results for the isolates were also generally in line with the kit interpretation table. Most of the isolates were generally able to metabolize simple sugars (glucose, ribose and sucrose – excepting maltose for some isolates) which are expected to be in abundance on the ocular surface. *C. propinquum* was different in this regard, being unable to metabolize the sugars tested. With few exceptions, the isolated *Corynebacterium* were unable to enzymatically hydrolyse aryl-substituted glycosides, but all hydrolysed the substituted phosphoester p-Nitrophenyl phosphate, indicating the presence of phosphatase activity. Several isolates showed urease activity. The test does not include a test for lipase activity but includes a test for esterases. The nature of the substrate is not specified beyond that it is a ‘fatty acid ester’.

### Deferred growth inhibition assay

**Fig. 1:**
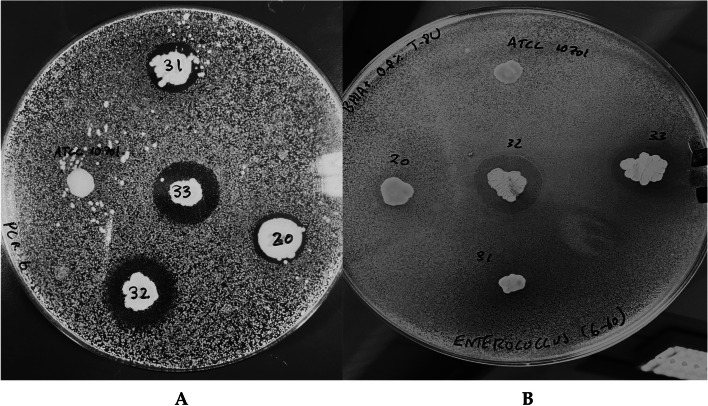
**A** Inhibition of a lawn of* C. sanguinis* PCR 6, (isolated from patient with mild dry eye [15]) by *Corynebacterium* sp. PCR 32 (replicates 32,33) isolated from a patient with moderate dry eye, and by *C. accolens* [20, 31] isolated from patients with severe and moderate dry eye respectively. Note that the control (ATCC10701, *C. pseudodiphtheriticum*) produces no zone of inhibition.** B** Inhibition of a lawn of *E.* *faecalis* (isolated from patient with severe dry eye) inhibited by *Corynebacterium* sp. PCR 32 (replicates 32,33) and *C. accolens* (PCR 20, PCR 31). No inhibition by the control ATCC10701 was seen

 The ability of the *Corynebacterium* isolates to inhibit the growth of some clinically relevant pathogens of the eye, as well as one another, was investigated using a deferred growth inhibition assay. All *Corynebacterium* isolates were tested for their ability to inhibit the growth of a panel of 3 test organisms (*P. aeruginosa* DSM22654, *C. bovis* (isolate PCR 37) and *S. aureus* DSM2569). In addition, a number of other inhibition tests were performed, particularly using test isolate *Corynebacterium* sp. PCR 32 which was in general the most effective isolate with respect to inhibition of the indicator bacteria. Supplementary Table 4 provides a summary of the results. *C. accolens* and particularly *Corynebacterium* sp. PCR 32 showed inhibitory activity against other ocular isolates including other *Corynebacterium* (Fig. 1A) and *Enterococcus faecalis* (Fig. 1B). Furthermore, it was found that the inhibitory agent(s) produced by *Corynebacterium* sp. PCR 32 was heat stable (supplementary Fig. 2). Additionally, *Corynebacterium* sp. PCR 32 exhibited a peculiar growth response when inoculated onto an agar plate where it was already growing (3 day growth). In brief, *Corynebacterium* sp. PCR 32 in low density inoculums would only grow about 5 mm from itself. No growth of the inoculums occurred closer to or further from the prior growth (see supplementary Fig. 3 and discussion in supplementary file).

### Whole genome sequencing (WGS) of *Corynebacterium*

In total, the genomes of 23 *Corynebacterium* isolates from DED sufferers and some controls were sequenced. These were comparatively small and in the size range previously reported for members of this genus [52]. Sequencing and genome metrics for each isolate, including the total number of high-quality reads obtained after sequence cleaning and filtering, mean read length, number of contigs and total genome size are summarized in supplemental Table 5. The GC content ranged from 55.5% in *Corynebacterium* sp. PCR 32 to 73% in *C. bovis* (PCR 38). The number of CDS predicted by RASTtk ranged from 2,044 in *Corynebacterium* sp. PCR 32 to 2,486 in *C. macginleyi* (PCR 7). The percentage of hypothetical proteins ranged from 43% in *C. bovis* (PCR i) to 26% in *C. marquesiae* (PCR 3). Plasmids were not detected in cellular lysate preparations of any of the isolates and this finding was supported by the WGS analysis.

### Speciality genes

**Fig. 2:**
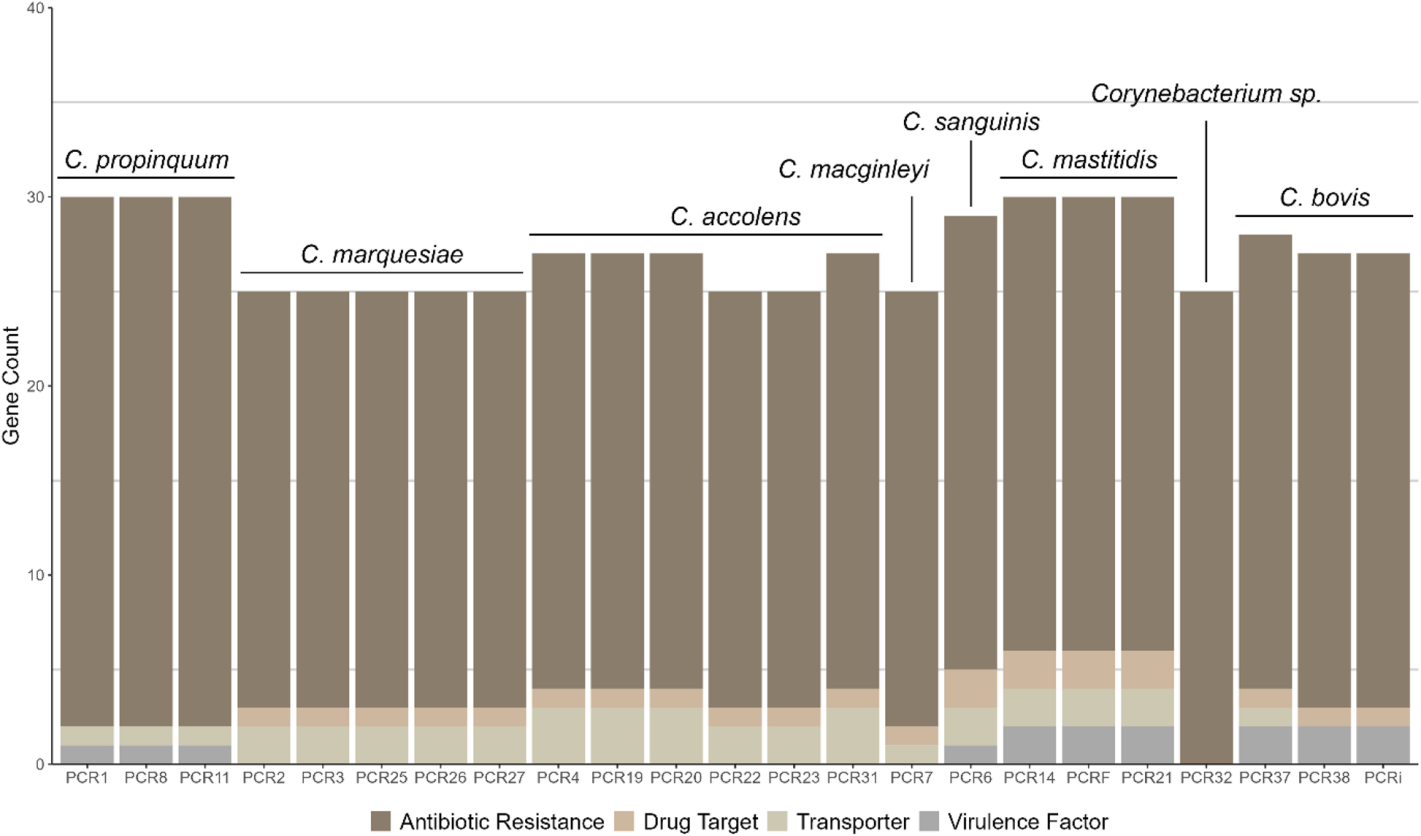
The distribution of antibiotic resistance genes, drug targets, transporter genes and virulence factors in the *Corynebacterium* isolates

The BV-BRC platform was used to perform a comprehensive genome analysis. The BV-BRC platform identifies so-called *Specialty genes* (antimicrobial resistance, virulence factors, drug targets and transporters) using BLASTP [41] to access a range of databases. CARD, NDARO and manually curated AMR genes were used as reference sources for antimicrobial genes. DrugBank and TTD were used to identify drug targets - i.e. known and explored therapeutic protein and nucleic acid targets. Virulence factors were mapped from VFDB, Victors database and from manually curated virulence factors. Transporter genes were mapped from TCDB. Identification of speciality genes was achieved using k-mer and BLAT. In summary, all isolates contained at least 20 different possible antibiotic resistance genes and at least 1 possible transporter gene. Drug targets were present in all isolates expect for *Corynebacterium* sp. (PCR32). Possible virulence factors were predicted to be present only in *C. marquesiae*,* C. sanguinis*,* C. mastitidis*, and *C. bovis* (Fig. 2). Salient details of the annotations for each *Corynebacterium* species are presented sequentially below.

#### *C.propinquum*

All 3 *C. propinquum* isolates (PCR 1, 8, 11) possessed one copy of the virulence factor isocitrate lyase. One transporter gene, arsenical-resistance protein ACR3, was found in each genome. No database drug target genes were detected. Each genome was found to have one copy of each listed antimicrobial target gene (supplementary Table 6) except for glycerophosphoryl diester phosphodiesterase (EC 3.1.4.46) which was present in two copies in all 3 isolates. Additionally, ResFinder identified the presence of the e*rm(X)*,* cmx* and *Sul1* resistance genes in all 3 isolates. These genes are associated with respectively resistance to Macrolides-Lincosamides-Streptogramins, Chloramphenicol and Sulphonamides.

#### *C.marquesiae*

All 5 *C. marquesiae* genomes (PCR 2, 3, 25, 26, 27) were identical in their specialty genes content, and contained no virulence factors listed in the VFDB or Victors databases. Of potential drug target genes, the isolates were found to have the RecA protein. The *C. marquesiae* isolates had both arsenical-resistance protein ACR3 and phosphate ABC transporter PstB (TC 3.A.1.7.1), classified as transporter genes. *C. marquesiae* were found to have 22 potential antimicrobial resistance targets (supplementary Table 6). In our previous study [15], these isolates were identified as the closely related *Corynebacterium tuberculostearicum* based on partial sequencing of the 16S rDNA and *rpo*B genes. ResFinder did not identify any potential resistance genes in the *C. marquesiae* genomes.

#### *C.accolens*

Six of the isolates (PCR 4, 19, 20, 22, 23, 31) included in this study were identified as *C. accolens* based on the WGS. None of the *C. accolens* isolates were found to contain any virulence factors, but all were found to have RecA protein, a possible drug target. Regarding transporter genes, all isolates contained the phosphate ABC transporter PstB (TC 3.A.1.7.1). Arsenical-resistance protein ACR3 was also present in all isolates. A hypothetical protein classified as a possible transporter protein was present in PCR 4, 19, 20 and 31. Lastly, 23 genes were found to possibly code for antibiotic resistance targets (supplementary Table 6). ResFinder identified the *erm(x)* gene associated with streptogramins in 4 of the *C. accolens* isolates (PCR 4, 19, 20, 31).

#### *C. macginleyi*

Based on WGS this isolate was found to be similar to *C. accolens*, but could only be reliably identified as a *Corynebacterium* sp. In our previous study [15], this isolate was found to be most like the species group *C. macginleyi-accolens* based on partial sequencing of the 16S rDNA and *rpo*B genes. In silico analyses using the TYGS pipeline [31] identify the isolate as *C. macginleyi* based both on dDDH values and the complete 16S rDNA sequence (see Figs. 4 and 5), and this identification is used throughout the current report. The isolate was found not to have any potential virulence factors listed in the Victors database or VFDB. It also contained the RecA protein and the phosphate ABC transporter PstB (TC 3.A.1.7.1). Possible antimicrobial targets found in the genome are listed in supplementary Table 6. Glycerophosphoryl diester phosphodiesterase (EC 3.1.4.46) was present in duplicate. ResFinder did not identify the *erm(x)* gene in the genome data.

#### *C.sanguinis*

The one isolate (PCR 6) which belonged to the *C. sanguinis* species contained the isocitrate lyase virulence factor. Of potential drug targets, the genome contained RecA protein and ribose-5-phosphate isomerase B (EC 5.3.1.6). The latter drug target was only present in this species among the ocular isolates. Additionally, two hypothetical proteins were found as potential transporter proteins. Antimicrobial targets present in this genome are listed in supplementary Table 6. ResFinder did not identify any potential resistance genes in the *C. sanguinis* genome.

#### *C. mastitidis *

The 3 is﻿o﻿lates identified as *C. mastitidis* (PCR 14, 21 and F) had identical specialty genes. All *C. mastitidis* genomes contained two virulence factors, namely isocitrate lyase and cAMP binding proteins, and two drug targets, RecA protein and naphthoate synthase. Two transporter genes, methionine ABC transporter permease protein and phosphate ABC transporter protein PstB (TC 3.A.1.7.1), were also found in all 3 isolates. Twenty-four potential antimicrobial targets were found in the isolates (supplementary Table 6). ResFinder did not identify any resistance genes in the 3 *C. mastitidis* genomes.

#### *Corynebacterium sp.*

Unlike the other *Corynebacterium* species, isolate (PCR 32) did not have any predicted virulence factors or potential drug targets. Specialty genes identified in this genome included 23 antimicrobial targets (supplementary Table 6). Of these, CDP-diacylglycerol–glycerol-3-phosphate 3-phosphatidyltransferase (EC 2.7.8.5) and glycerophosphoryl diester phosphodiesterase (EC 3.1.4.46) were present in duplicate. The genome of the isolate was the smallest of the ocular *Corynebacterium.* ResFinder did not identify any potential resistance gene in this genome.

#### *C. bovis*

All 3 isolates identified as *C. bovis* (PCR 37, 38, i) were identical in terms of their specialty genes, except that PCR 37 contained an additional transporter gene (phosphate ABC transporter ATP-binding protein PstB (TC 3.A.1.7.1)). Two potential virulence factors, isocitrate lyase and cAMP binding proteins and 1 drug target, RecA protein, were present in all 3 isolates. In all, 24 potential antimicrobial targets (supplementary Table 6) were also found to be present in all 3 isolates. No additional resistance genes were identified by ResFinder.

### Pangenome of the *Corynebacterium* isolates

Our analysis of the pangenome (the core, accessory, and unique genes present) of the isolates, showed that the core genome consisted of 516 genes, whereas the number of accessory genes ranged from 419 (*Corynebacterium* sp. PCR 32) to 1843 (*C. accolens*). The pangenome profile analysis with BPGA is shown figuratively in supplemental Fig. 4 and illustrates the total number of shared and distinct gene families in each isolate. The addition of genomes to the analysis resulted in an increase in the number of gene families suggesting that the pangenome is open. This observation has been reported previously in *Corynebacterium* species [29].

**Fig. 3:**
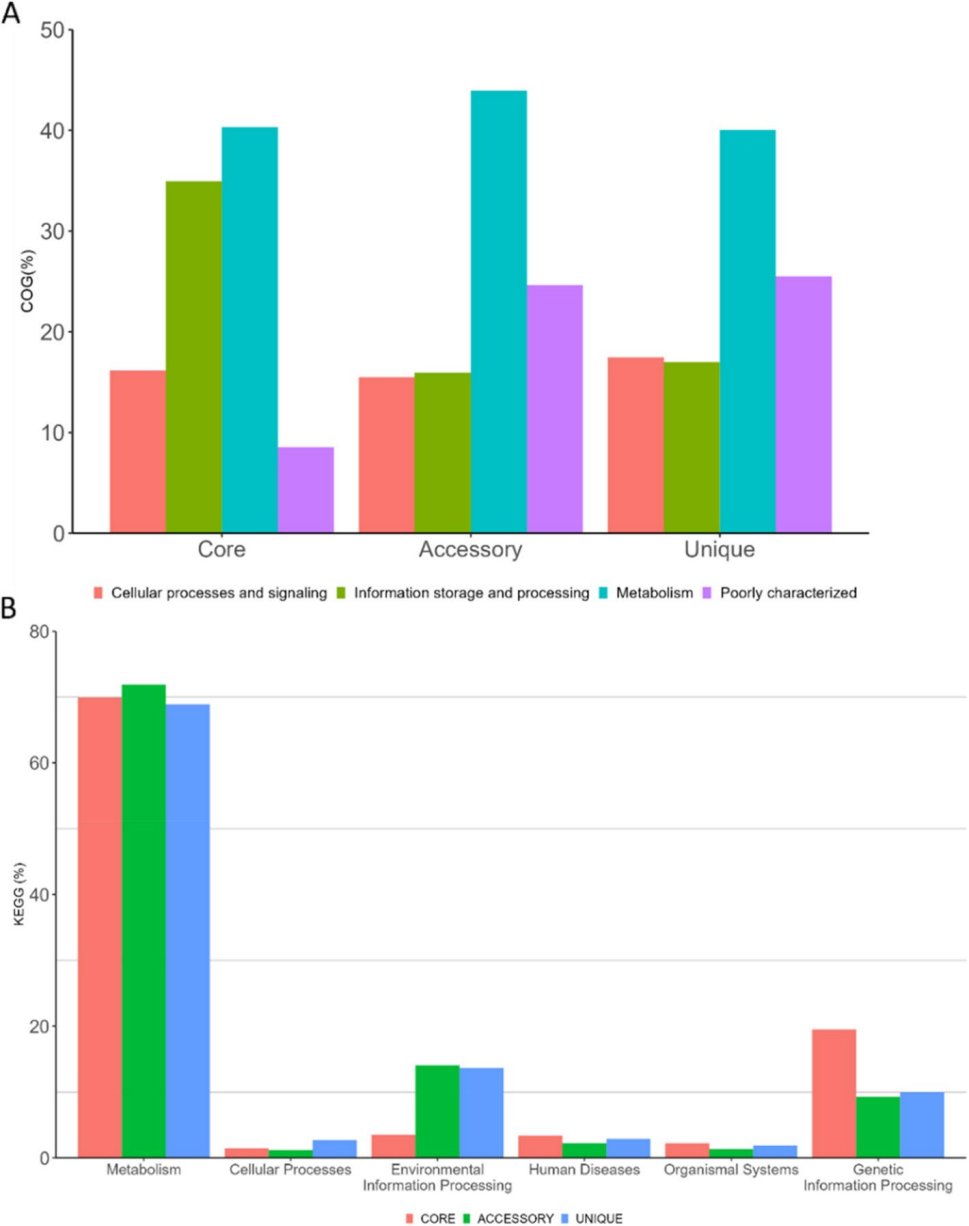
Clustering of orthologous genes (**A**) and their distribution (**B**) in the core, accessory, and unique genomes

We further conducted clustering of orthologous genes (COG) and KEGG analysis of the core, accessory, and unique genomes (Fig. 3A and B). Fourty % of the core genome was found to be involved in metabolism and 34% in information storage and cellular processing. The same trends were observed for both the accessory genome and the unique genomes, in which 43% and 40%, respectively of the genes contributed to metabolism. The largest difference between the unique and core genomes was seen with poorly characterized genes (25% and 8%, respectively). The 3 genome categories were found to contribute significantly to metabolism (core, 70%; accessory, 72%; and unique, 69%). A difference between the genome categories was observed in the percentage of genes which are involved in genetic information processing. Here the core genome contains 19.5%, whereas for the accessory and unique genomes this value was 9%. Conversely, a higher % was found in the environmental information processing category, in which the accessory genome contributes with 14% and the core genome with 3%, respectively. It should be noted that the KEGG analysis only accounts for annotated genes, thus the % of the KEGG categories for the unique genome may differ as a larger proportion of the proteins are poorly characterized.

### Phylogenomic analyses and phylogenetic trees

A number of different metrics can be used to distinguish between closely-related species such as average nucleotide identity (ANI), DNA-DNA hybridization and difference in GC% in the genomes of interest [31]. It has been suggested that that isolates with an ANI > 95% can be considered to be the same species [53]. Furthermore, isolates belonging to the same species usually have less than 1% differences in their GC content [31]. TYGS and the JSpeciesWB tool were used to calculate these metrics and create phylogenetic trees for PCR 32 as well as the isolate identified as *C. macginely* (PCR 7) where the WGS were inconclusive with respect to their identities. Phylogenetic trees were inferred with FastME from GBDP distances calculated from either the 16 S rDNA gene sequences or the genome sequences.

#### *C*. *macginleyi*

**Fig. 4:**
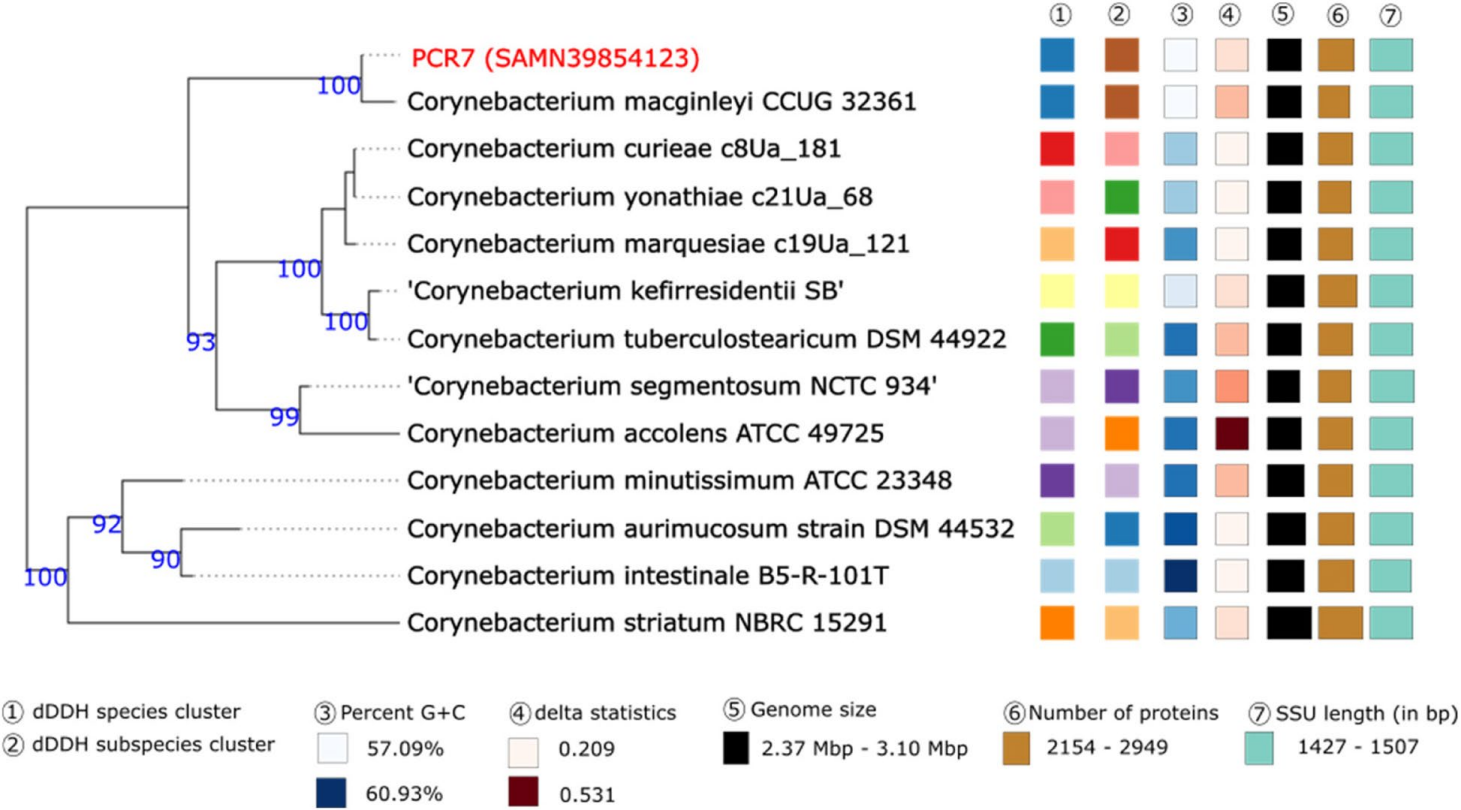
Phylogenetic tree created based on the 16S rDNA gene sequences using the GBDP distance formula d5 to calculate the branch lengths. The numbers above branches are GBDP pseudo-bootstrap support values > 60% from 100 replications, with an average branch support of 85.0%. The tree was rooted at the midpoint and inferred with FastME. Red labels indicate isolates from this study

**Fig. 5:**
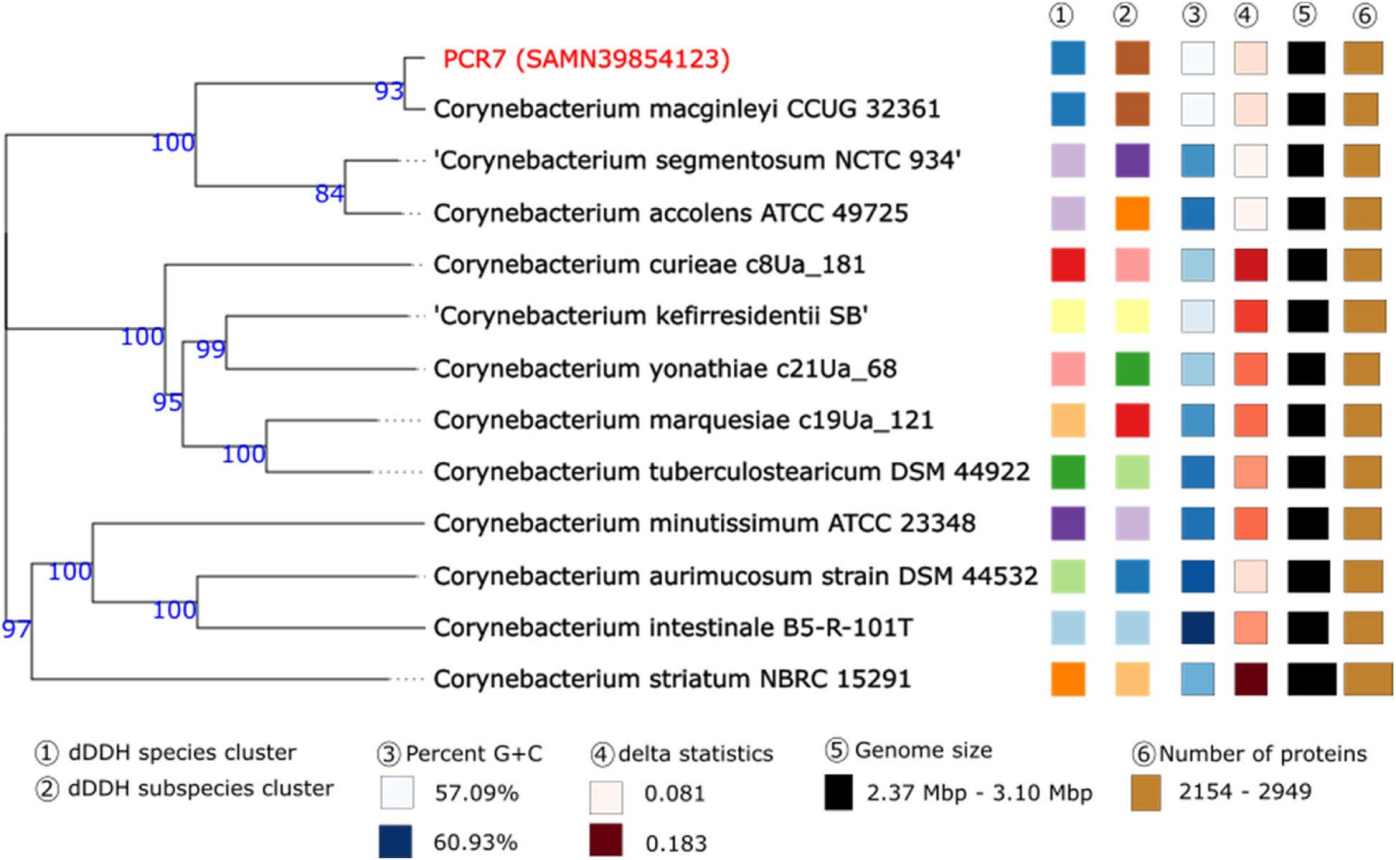
Tree inferred with FastME 2.1.6.1 from GBDP distances calculated from genome sequences. The branch lengths are scaled in terms of GBDP distance formula d5. The numbers above branches are GBDP pseudo-bootstrap support values > 60% from 100 replications, with an average branch support of 96.8%. The tree was rooted at the midpoint

PCR 7 was uploaded to the TYGS platform for phylogenetic analyses using the 16S rDNA gene and genome sequences (Figs. 4 and 5). Both trees illustrate the close relationship between PCR 7 and *C. macginleyi.* The dDDH values of PCR 7 and *C. macginleyi* CCUG 32,361 were 90.4% [CI 88.1–92.3] and the difference in the GC% was 0.08%. In addition, the ANI value between the genomes was 98.73%. The three abovementioned metrics as well as the phylogenetic trees indicate that this isolate is *C. macginleyi.*

#### *Corynebacterium* sp.

**Fig. 6:**
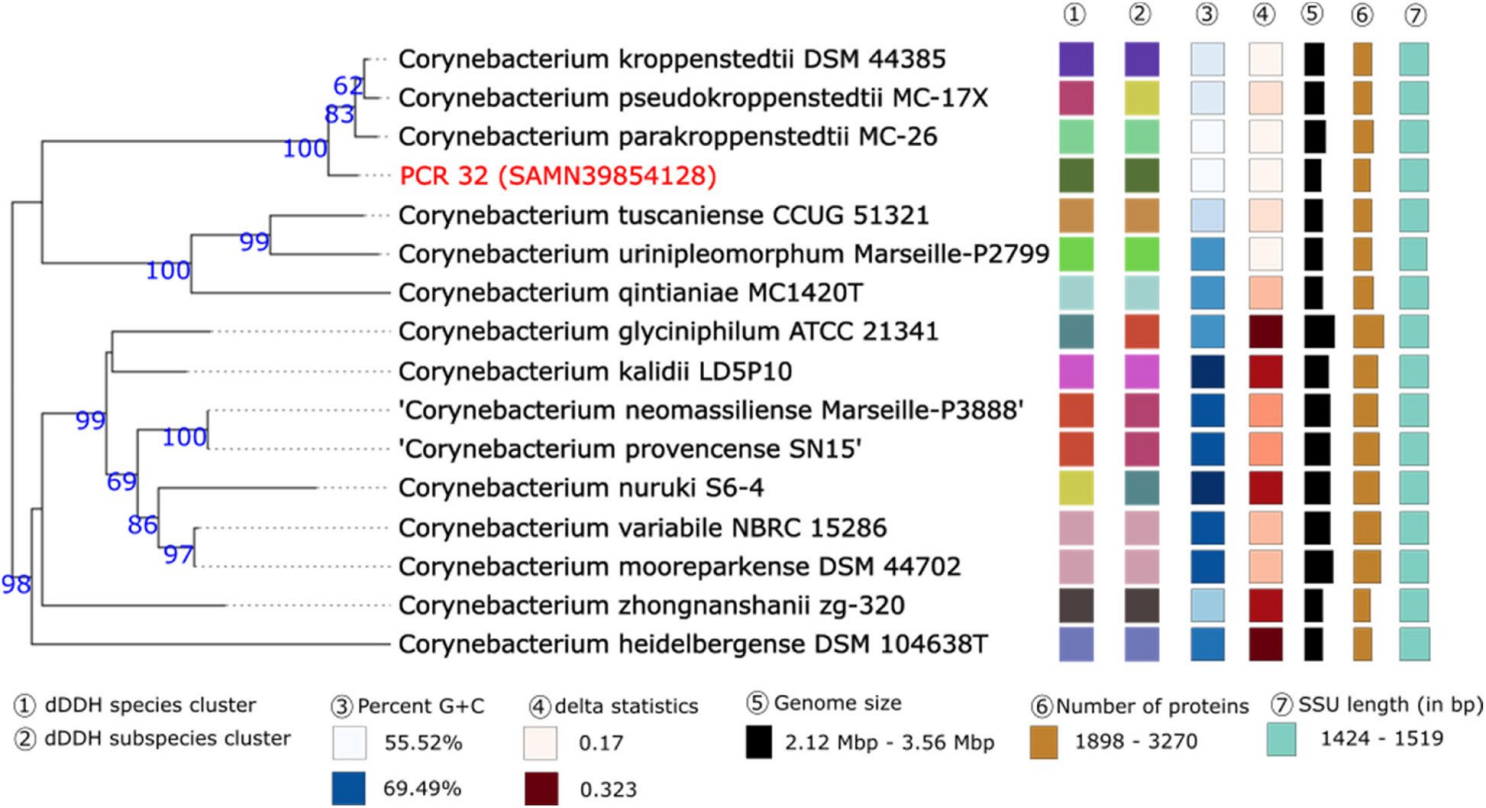
Phylogenetic tree based on the 16S rDNA gene sequences was inferred with FastME. The GBDP distance formula d5 was used for branch scaling. The numbers above branches are GBDP pseudo-bootstrap support values > 60% from 100 replications, with an average branch support of 85.4%. The tree was rooted at the midpoint

**Fig. 7:**
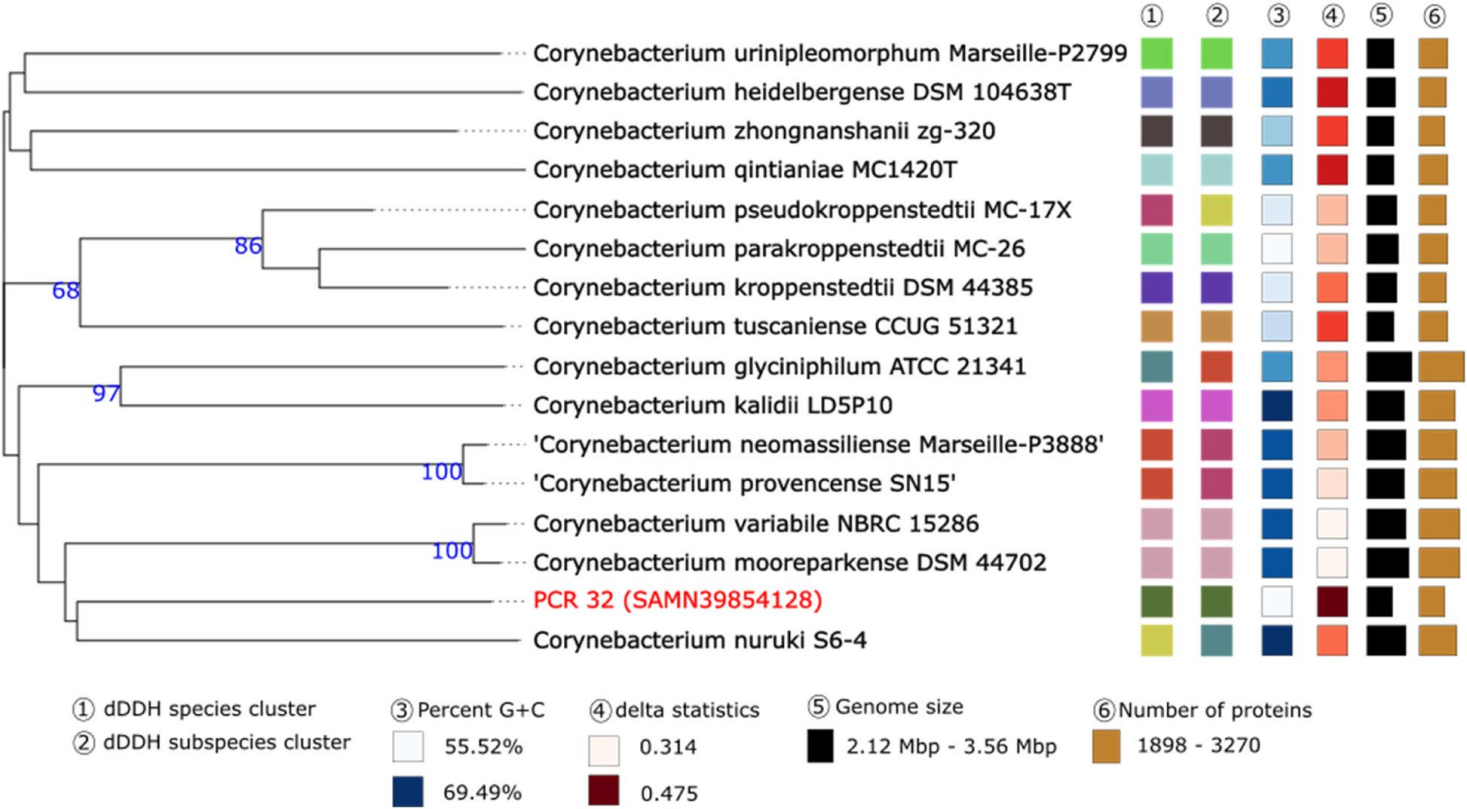
Tree inferred with FastME 2.1.6.1 [7] from GBDP distances calculated from genome sequences. The branch lengths are scaled in terms of GBDP distance formula d5. The numbers above branches are GBDP pseudo-bootstrap support values > 60% from 100 replications, with an average branch support of 53.5%. The tree was rooted at the midpoint

Isolate *Corynebacterium* sp. PCR 32 could only be reliably identified as *Corynebacterium* sp. based on WGS. Therefore, additional in silico tests were performed. Phylogenetic trees using the 16S rDNA gene indicated that this isolate could be a part of the *C. kroppenstedtii* complex [54] (Fig. 6). Phylogenetic trees created using genome sequences it placed this isolate in a poorly-supported clade with *C. nuruki* (Fig. 7). The dDDH value between PCR 32 and *C. parakroppenstedtii* MC-26 was 20.0% [CI 17.8–22.4] with a GC% difference of 1.35. The dDDH value between *C. nuruki* S6-4 was 23.6% [CI 21.3–26.0] and the GC% difference was 13.98%. ANI calculations between PCR 32 and *C. parakroppenstedtii* MC-26 and *C. nuruki* S6-4 were 75.19% and 67.60% respectively. These data collectively indicate that PCR 32 is possibly a new species.

### Lipase and Lipase-like sequences of *Corynebacterium*

**Fig. 8:**
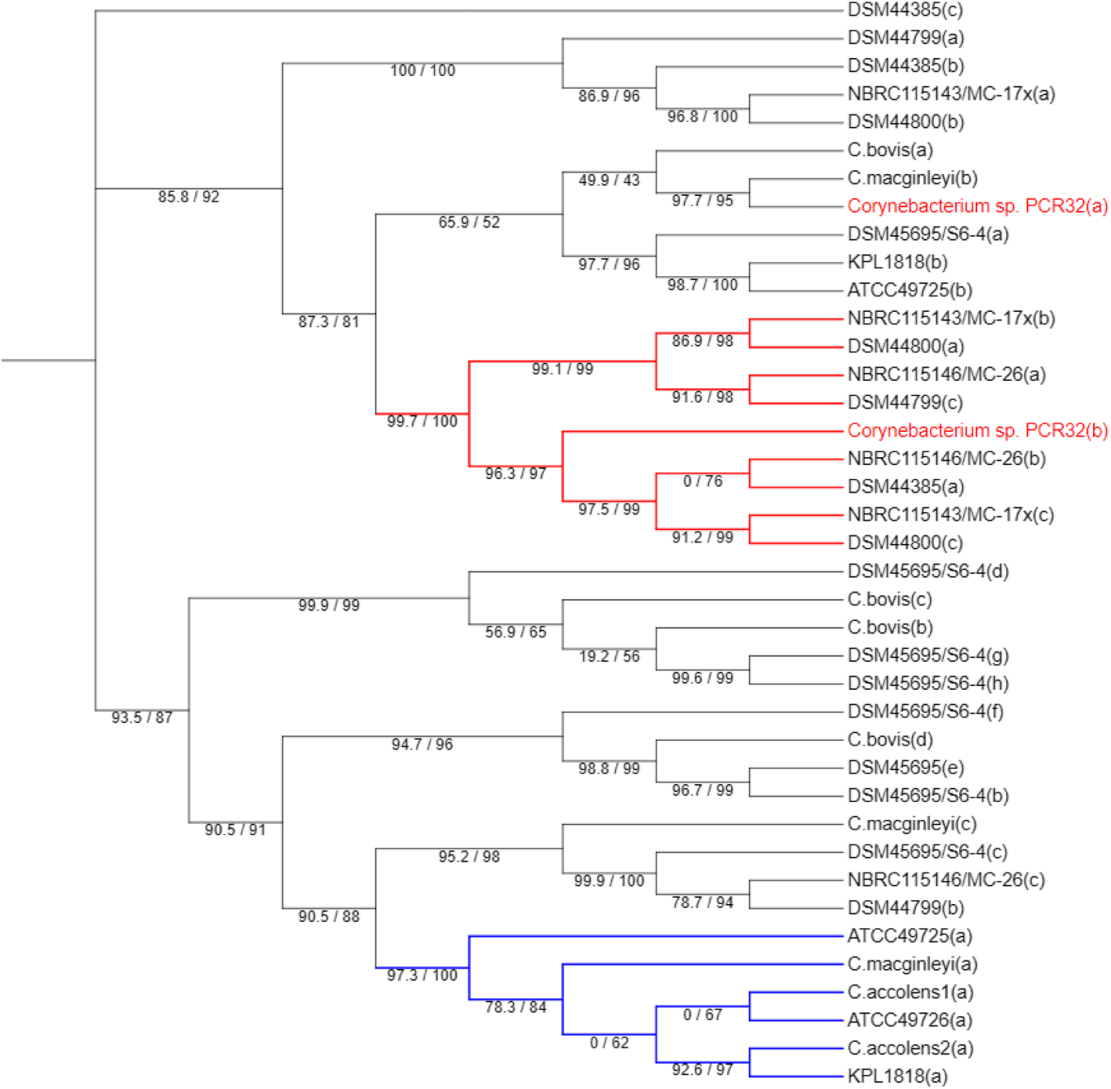
Maximum likelihood phylogenetic tree of lipases and lipase-like enzymes inferred using IQ-tree with the best-fit portioning strategy (WAG + F + I + G4). Branch support indicated at nodes: ultrafast bootstrap approximation/SH-like approximate likelihood ratio test results (1000 replicates). For isolates from the current study the species name is given. For strains included for purposes of comparison, the culture collection identification code is given. Lower case letters (a, b, c etc.) indicate different lipases. *Corynebacterium* isolates: *C. accolens* PCR20 (1(a)) and PCR22 (2(a)). *C. macginleyi* PCR7 (a, b, c). *Corynebacterium* sp. PCR32 (a, b) and *C. bovis* PCR37 (a, b, c, d). Culture collection strains: *C. kroppenstedtii*^T^ DSM44385, (a, b, c); *C. kroppenstedtii* DSM44799 (a, b, c); *C. kroppenstedtii* DSM44385 (a, b, c); *C. kroppenstedtii* DSM44800 (a, b, c). *C. pseudokroppenstedtii* NBRC115143T/MC-17x (a, b, c). *C. parakroppenstedtii* NBRC115146T/MC-26 (a, b, c). *C. nuruki* DSM45695T/S6-4 (a-e). *C. accolens* ATCC49725T (a, b); *C. accolens* ATCC49726 (a). *C. accolens* KPL1818 (a, b): sequence (a) is a triacyl glycerol lipase (TAG lipase) shown by Bomar et al., [12] to cleave the TAG triolein releasing oleic acid which inhibited growth of *Streptococcus pneumoniae.* Accession numbers for the genomes are given in supplementary Tables 1 and the alignment file for the tree are in the supplementary file 1

Growth inhibitory effects of *C. accolens* have previously been reported and were attributed to TAG lipase activity [12]. Figure 8 shows the cladogram of the maximum likelihood phylogenetic tree from alignment of lipase and lipase-like *Corynebacterium* sequences taken from GenBank with those from the present study for two *C. accolens* (PCR 20 and 22), *C. macginleyi* (PCR 7), *Corynebacterium* sp. (PCR 32) and *C. bovis* (PCR 37) isolates. The isolates were chosen for further analysis based mainly on lipase-activity (+ or -) (Table 1 and supplementary Table 3) and results of the growth deferred assay (effect/no effect) (supplementary Table 4). Other lipase sequences obtained from GenBank for inclusion in the tree were chosen based on the criteria described in the materials and methods. The figure legend provides additional details on the included sequences and model parameters for tree topology.

A lipase of *Corynebacterium* sp. PCR32 (lipase b) forms part of to two well-supported groups of clades (coloured red) made up of exclusively lipases from reference strains of *C. kroppenstedtii*, *C. parakroppenstedtii* and *C. pseudokroppenstedtii*. These enzymes showed variously the presence of a signal peptide, and none contained the canonical G X S X G sequence of lipases. A possible alternative motif GNSAR was found in all. To provide further in silico proof of the functional nature of the *Corynebacterium* sp. PCR32 (a) and (b) enzymes, the recently developed PhiGnet AI tool [55] which predicts protein function using statistics-informed graph networks was used. The pipeline is available online at (https://kornmann.bioch.ox.ac.uk/jang/services/phignet/index.html). The analysis returned an identification of triacyl glycerol lipase E.C.3.1.1.3 with a confidence of > 0.998 for both enzymes. *Corynebacterium* sp. PCR 32 (a) showed only low sequence identity (< 40%) with other sequences. It contained the canonical G X S X G motif, but not a signal peptide. *C. accolens* isolate lipases (1a, 2a) and a lipase of the closely related *C. macginleyi* clustered (clade shaded blue in Fig. 8) with high sequence identity (89–100%) with those of putative TAG secreted lipases in the annotated genomes of members of the *C. accolens* species (Interpro family IPR005152 = ‘secreted lipase’). This clade included KPL1818(a) previously shown to be associated with growth-inhibitory effects of *S. pneumoniae* [12].

Three lipase sequences (b, c, d) of *C. bovis* PCR 37 (which tested as lipase-negative and did not inhibit any indicator strains) had both probable export signal peptide sequences and the canonical G X S X G of secreted lipases.

## Discussion

The aim of this study was to characterize *Corynebacterium* species isolated from the ocular surface. Twenty-three isolates representing 8 species from both DED sufferers and healthy eyes were included [15]. To our knowledge no previous reports of WGS and phenotypic characterization of ocular *Corynebacterium* isolates exist. This makes the present study important, given that members of this genus can both form a significant part of the healthy core ocular microbiome [9], but can also rise in abundance to dominate (> 90% of total reads) NGS libraries of DED patients and occasionally controls [15]. The latter instances would be considered to represent a dysbiosis in the ocular microbiome, and as such may have a negative impact on ocular surface health. A further point of importance in relation to ocular health, is that DED is typically characterized by a chronic inflammation which potentially could be related to the numbers and types of microbes present [56]. The majority of ocular microbiome studies report the presence of *Corynebacterium* on the ocular surface, and often conclude that the genus is a commensal with possible protective mechanisms [10, 57]. In contrast, other studies suggest that *Corynebacterium* can be an infectious agent resulting in keratitis, corneal ulcers or conjunctivitis [7], or be associated with meibomian gland dysfunction which is one of the most common forms of DED [58, 59]. It is unclear under which circumstances and which members of the genus are beneficial or impacting negatively on ocular health. The present study attempts to shed light on this. To characterize the *Corynebacterium* isolates we performed both WGS and/or phenotypic tests on bacteria previously isolated from ocular swabs [15].

WGS of the isolates showed that all isolates had relatively small genomes ranging from 2.12 to 2.65 Mbp as has been previously reported for the genus [6, 29]. This is particularly interesting considering that pathogenic bacteria tend to have smaller genomes than their nonpathogenic relatives [60]. This observation has also been reported in a previous study on pathogenic and non-pathogenic *Corynebacterium* [29]. The presence of a small genome is not necessarily a direct indication of pathogenicity, but does illustrate the capacity of the microbe to acquire new potential virulence genes [61]. Moreover, analysis of the pangenome indicated that it is open (supplementary Fig. 4), as has been previously reported for the genus [29].

WGS of the *Corynebacterium* isolates also indicated the presence of a range of specialty genes which included potential antibiotic targets, drug transporters, virulence factors and transporter genes. Potential antibiotic targets for each isolate are listed in supplementary Table 6. RecA was the most common drug target found in the isolates, and isocitrate lyase, which is involved in the glyoxylate cycle, was the most common virulence factor identified. Isocitrate lyase has previously been shown to be essential for a range of bacteria for virulence in a range of bacterial types [62].

Antibiotic treatment is sometimes indicated for DED [63] and given that overgrowth of *Corynebacterium* can occur on the ocular surface, it was pertinent to look at the susceptibility properties of the isolates. The isolates were susceptible to several ocular treatment relevant antibiotics such as fluoroquinolones. Aoki et al., [7] report the use of fluoroquinolones as first choice treatment of *Corynebacterium* ocular infections in Japan, and that resistance is a problem. All of the *Corynebacterium* isolates in the present study showed intermediate susceptibility to ciprofloxacin and complete sensitivity to moxifloxacin. The same authors report the use of vancomycin in treatment of ocular infections and all isolates in the present study showed complete sensitivity to this antibiotic. ResFinder identified *erm(X)*,* cmx and Sul1* in several isolates. As no breakpoint for erythromycin is currently available for the *Corynebacterium* isolated in the present study it was not included in supplementary Table 2. However, we noticed that *Corynebacterium* possessing *erm(X)* gene gave no zones of inhibition around discs containing 15 µg erythromycin (results not shown). Likewise, the *C. propinquum* isolates which also had the *erm(X)* gene had much narrower zones of inhibition for erythromycin compared to other *Corynebacterium*. Two of the isolates (*C. marquesiae* PCR 3 and 27) were resistant to 3 antibiotics belonging to 3 different antimicrobial categories, and would according to current proposed definitions be considered as multidrug resistant [64]. The MDR definition adopted is that proposed by European Centre for Disease Prevention and Control (ECDC) and the Centers for Disease Control and Prevention. Other definitions exist, but a review of these is beyond the scope of the present study. Although multiresistance was shown, these isolates were completely sensistive to other clincially relevant antibiotics (supplementary Table 2). Given the demonstration of multiresistance, it is important to monitor resistance development in this species.

Given the close proximity of their sites of origin, ocular *C. accolens* may once have originated from the more heavily populated nasopharynx or vice versa. A side-by-side analysis of speciality genes and phenotypic features would be required to see which, if any, adaptations to their particular bodily niches have occurred. It was found that in general *Corynebacterium* isolates belonging to the same species from the same patient were highly clonally related. *C. accolens* PCR 4, 19, 20 and 31 were highly similar with an ANI of 99.9%. This group of isolates was collected from the same dry eye patient (Table 1). This suggests that a single clone may have established itself and proliferated on the ocular surface which would be in line with our previous report of large single species populations on the eyes of DED patients [15].

Phylogenomic analyses of the *Corynebacterium* sp. PCR 32 including dDDH and ANI values suggest the isolate may require re-classifications as a separate species. The isolate formed a well-supported clade with the type strains of *C. kroppenstedtii* complex (*parakroppenstedtii*, *pseudokroppenstedtii* and *kroppenstedtii*) but based on the ANI and dDDH values cannot be considered to be a member of this complex. Further studies will be required to define precisely the taxonomic placement of the isolate.

Some extracellular enzyme activities often connected to colonization and pathogenicity in microbes were tested for (Table 1 and supplementary Table 3). These included lipases, DNase, protease, mucinase and urease. Activities of each enzyme specificity were found in one or more isolate. *Corynebacterium* sp. PCR 32 showed definite (+) or possible (+/-) activity for 4/5 enzyme classes tested for. *Corynebacterium* sp. PCR 32 was unique among the isolates in showing hydrolysis of skimmed milk proteins which are chiefly casein (Table 1). There are several potential candidates for this activity in the annotated genome, chief of which is a sequence with a putative secretory signal peptide showing about 68% identity with a trypsin-like serine protease.

*C. propinquum*, which has been associated with keratitis [7] was unique in the production of extracellular DNase activity. DNases have been implicated as virulence factors in a variety of situations ranging from enhanced bacterial growth and biofilm maturation to the ability of bacteria to escape the immune system [65]. DNase activity in the pathogenic *Corynebacterium* species, *C. diphtheriae* and *C. ulcerans* has been described as a virulence factor for many decades [66].

There were also (weak) indications of mucinase activity for several isolates (Table 1). Mucin degradation is considered a part of the normal turnover of mucus layers, but mucinase activity is also a virulence factor in the invasion of tissues including the ocular surface. Mucins are present at the ocular surface in both secreted and membrane-bound forms. In the tear film, they play a role in lubrication and ocular defense, functioning as a barrier that protects the eye against damage and infection [67]. A wide-variety of enzyme activities can be involved in mucin degradation (including glycosidases, proteases, and sulphatases) making identification of the underlying genetic determinants of the weak activity recorded challenging. *C. accolens* and *Corynebacterium* sp. PCR 32 showed mucinase activity (Table 1) and as shown previously by our group, were among those species able to reach high percentage abundancies (> 90%) on the ocular surface [15]. At such high bacterial abundancies, mucinase activity may negatively impact on protective ocular barriers. However, this will require further investigation.

The isolates were characterized with respect to the utilization of 18 different biochemically substrates (supplementary Table 3). Of these especially urease activity is considered relevant for ocular health. Several *Corynebacterium* ocular isolates were urease producers. It has previously been shown that a number of pathogens such as *Corynebacterium urealyticum*, where urease activity has been associated with the formation of renal stones [68], are able to utilize urea as a nitrogen source through the activity of urease. The reactions involved ultimately lead to an increase in local pH that can interfere with host cell function. Enzymes of urea synthesis are expressed at the ocular surface, and decreased urea in the tear fluid is significantly associated with DED [69].

Of special interest were the results of the deferred growth inhibition assay. Several species (especially *Corynebacterium* sp. PCR 32) were shown to have antibacterial effects on both ocular pathogens (such as *S. aureus* and *P. aeruginosa*), as well as other ocular *Corynebacterium.* Among the isolates showing antibacterial activity were *C. accolens* and *C. macginleyi* (see Fig. 1 and supplementary Table 4). In our previous NGS analysis of ocular isolates based on partial sequencing of the 16 S rRNA gene [15], some sequences were classified by the Zymo Research^®^ 16S curated, proprietary database as *C. accolens-macginleyi*. These are presumably sequence reads that were incompletely resolved taxonomically owing to the two species being very closely related. In some instances, this sequence group was found to be able reach high percentage abundances (as high as 99% in one DE patient) in the NGS libraries. One potential explanation for this, which would require more targeted approaches to confirm, is that these bacteria are somehow outcompeting other bacteria present on the ocular surface. As we used both culture and non-cultured based approaches in our previous study [15] we were able to perform WGS of ocular isolates and found isolates of both *C. accolens* and *C. macginleyi* (this study). Bomar et al. [12] showed that *C. accolens* is able to inhibit the growth of other bacteria, including *S*. *pneumoniae*, and the co-authors were able to attribute this to the production of an exported TAG lipase (Fig. 7 KPL1818(a)). It is thus possible that extracellular enzyme activities, particularly lipase, may explain the results of the deferred growth inhibition assay for the other *Corynebacterium* present in this study. Indeed, both *C. accolens* and *C. macginleyi* ocular isolates possessed a lipase showing a high degree of sequence identity and similar motifs to KPL1818(a) (Fig. 7 and supplementary file 1). The profound growth inhibitory effects (Fig. 1, supplementary Table 4) seen in vitro with *Corynebacterium* sp. PCR32 do not as easily fit into this hypothesis, as no KPL1818(a) type TAG lipase was found in the genome. This apparent *discrepancy* is discussed in more detail in the following section and in the Supplementary data of this report. Although there is evidence from *C. accolens* that lipase activity freeing long chain, antimicrobial fatty acids is one mechanism of growth inhibition, almost certainly other mechanisms can be involved. Menberu et al., [13] used the deferred growth inhibition assay (with Tw80-containing agar) to look for antistaphylococcal effects of *C. accolens* isolates. The co-workers found variously no to strong effects depending on both the isolate and the indicator strain of *S. aureus*. However, they also demonstrated that concentrated culture supernatants had direct antistaphylococcal effects, and that this activity could be abolished by treatment with proteinase K; a finding which suggested the involvement of other factors than lipid hydrolysis. This is a finding that could fit the mode of for example bacteriocin activity. Therefore, other potential determinants of growth inhibition, particularly bacteriocins were looked for in the genomes (see supplementary data for a more in-depth discussion of this theme). To summarize, we identified various putative genetic determinants of lipolytic activity in *Corynebacterium* sp. PCR32, *C. accolens* and *C. macginleyi*, which showed growth inhibitory activity, as well as *C. bovis* which did not. The growth inhibitory activity of *C. accolens*, *C. macginleyi* and *Corynebacterium* sp. PCR 32, would be most easily explained by reference to their hydrolysis of esterified fatty acids by an SP18188(a) type TAG lipase, producing inhibitory long chain organic acids from tw-80, as previously described for *C. accolens* [12]. However, as mentioned above other than for the *C. accolens* and *C. macginleyi* isolates it was difficult to ascribe the genetic determinant of this activity in the genome. Similar functions might, however, be ascribable to other lipase-types found in the genomic material of other species (see further information in supplementary file). Whatever their nature, the antibacterial factors indicated by the deferred growth assay could it is speculated, be contributing to dysbiosis in some patients where *Corynebacterium* DNA reads come to dominate. They could conceivably also be drivers of ocular inflammation seen in DED patients [58]. These are avenues of research which we feel could be profitably followed.

### *Corynebacterium* as probiotics?

Owing to inhibitory effects on pathogen growth, the use of *Corynebacterium* as probiotics in connection with vaginal [70], intestinal [16] and nasopharyngeal [11–13] sites has been raised. However, perhaps a word of caution should be sounded here arising from our previous studies on the ocular microbiome [15]. In their review of the topic, Aoki et al., [7] noted that *Corynebacterium* species can be found commonly on the ocular surface, where they act together with other commensals such as *Staphylococcus epidermidis* and *Cutibacterium acnes* to protect the eye from invasion by foreign organisms. However, *Corynebacterium* are also frequently associated with a range of ocular infections. Aoki er al., [7] use the example that *C. macginleyi* is the most commonly isolated strain in the conjunctiva, but it is also recognized as the most common causative agent of opportunistic ocular infections. In our previous study, averaging over 61 patients with varying degrees of dry eye disease and 30 controls. We found that in about 12% of samples, and including both patients and controls, single *Corynebacterium* sequence reads (‘one species’) reached over 75% of the total and a variety of species including *C. accolens-macginleyi* and *C. kroppenstedtii* could dominate. In two striking instances, patient 16S rDNA libraries were dominated (95–99%) by a sequence typing as *C. accolens-macginleyi* (see discussion above on the use of this name). This outcome could be explained by a rise to dominance of a single *Corynebacterium* clone and in such a dysbiosis a protective level of other and perhaps beneficial microbes could be abolished, predisposing to infection with pathogens. Instances of high single-sequence percentage abundancies included but were not restricted to species demonstrated to have antibacterial effects in the present study. For example, in a young female patient with severe DED, 81% of sequence reads were *Corynebacterium* of which 80.5% were *C. bovis*. *C. bovis* did not inhibit the growth of other bacteria (supplementary Table 4) and generally did not produce hydrolytic enzymes (Table 1) associated with pathogenicity. The dysbiosis seen with this *Corynebacterium* species could then potentially be attributable to complex mechanisms beyond simple production of antimicrobials.

In this study, we present the whole genome sequences as well as their phenotypic characteristics of 23 *Corynebacterium* isolates from ocular samples. We isolated 8 different species from the ocular surface of DED patients as well as some healthy controls. Although, none of the isolates appeared to be primary pathogens our analysis detected lipase, mucinase, protease, urease, and DNase activity. These enzymes can be important factors for the colonization antagonism of the ocular surface. Furthermore, we detected the activity of antibacterial agents by some isolates of which lipases are hypothesized as the most likely agents. The exact nature of these awaits confirmation, but they could potentially be involved in both *Corynebacterium* dominated dysbiosis seen in some patients and be drivers of ocular inflammation characteristic of the DED state. Future work currently under planning will use differential transcriptomics to study how indicators and test strains respond to one another in the deferred growth inhibition assay. This will enable us to pinpoint upregulated genes and perhaps provide clues as to the exact nature of the antimicrobial agent(s).

## Supplementary Information

Additional file 1. Whole genome sequencing and characterization of *Corynebacterium* isolated from the ocular surface of dry eye disease sufferers.

## Data Availability

The datasets generated and/or analyzed during the current study are available in the GenBank repository with the following BioProject number: PRJNA1074235.

## Abbreviations

ANI: Average Nucleotide Identity
AST: Antimicrobial Susceptibility Testing
BHIA: Brain Heart Infusion Agar
BV-BRC: Bacterial and Viral Bioinformatics Resource Centre
COG: Clusters of Orthologous Genes
DED: Dry Eye Disease
KEGG: Kyoto Encyclopedia of Genes and Genomes
TAG: Triacylglycerols
Tw80: Tween-80
WGS: Whole Genome Sequencing
