# Supplementary material for: Whole genome sequencing and characterization of Corynebacterium isolated from the healthy and dry eye ocular surface
Source: BMC Microbiol. 2024 Sep 28;24:368. doi: 10.1186/s12866-024-03517-9 (PMC11438203; doi:10.1186/s12866-024-03517-9)
Supplement: Supplementary file 1 — Additional file 1. Whole genome sequencing and characterization of Corynebacterium isolated from the ocular surface of dry eye disease sufferers. [file 12866_2024_3517_MOESM1_ESM.docx]

**Whole genome sequencing and characterization of *Corynebacterium* isolated from the ocular surface of dry eye disease sufferers**

Supplementary materials and methods

2.1 Skimmed milk agar (protease activity)

Agar plates were prepared as follows. A solution of 10% skimmed milk powder (Sigma-Aldrich, Merck, 70166) was prepared in water. After autoclaving and cooling to 50°C, 50 ml of the milk was added with mixing to 450 ml autoclaved R_2_A agar (Oxoid, Hampshire, UK) previously amended with 0.01% Tween-80 before pouring. After inoculation, plates were scored for zones of complete hydrolysis around the bacterial growth. *Pseudomonas aeruginosa* ATCC 15692 was used as a positive control organism.

2.2 Olive oil-Rhodamine agar (lipase activity)

R_2_A agar (Oxoid, Hampshire, UK) was amended with highly refined olive oil (Sigma-Aldrich, O1514) as follows: 30 mL of olive oil was emulsified (Vibra-Cell VCX130, Sonics) into 50 mL water containing 250 µL of Tween 20 (Sigma-Aldrich, St. Louis, MO, USA, P1379). The emulsion was autoclaved, cooled to 60 °C and amended with 20 mL of a filter sterilized Rhodamine B solution (1 mg/mL). Then, 50 mL of this mixture was added with stirring into 450 mL R2A agar melted at 50°C. After inoculation and incubation, plates were examined for orange, fluorescent zones emanating from around colonies and/or zones of clearing around colonies developing over a period of 1 week - either or both of these growth effects were taken as an indication of lipase activity. *Burkholderia cepacia* DSM9421 and *Pseudomonas aeruginosa* DSM 1128 were used as positive control organisms.

2.3 DNA agar (DNase activity)

Commercially available plates containing 0.2% w/v deoxyribonucleic acid (Oxoid, Hampshire, UK, Catalogue number CM0321) were used. After incubation for 1 week, plates were flooded with 1N HCl and allowed to stand for 5 min. Zones of clearing in the agar around colonies was taken to indicate DNase activity. *Staphylococcus aureus* strains ATCC 25923 and DSM 799 were used as positive control organisms.

2.4 Porcine stomach-mucin agar (mucinase activity)

Mucin from porcine stomach (Sigma-Aldrich, Merck M2378) was included at 0.5% w/v in brain heart infusion agar (CM1136B, ThermoFisher scientific) and in R_2_A agar. Tween-80 at 0.01% w/v was also added to each agar type. The growth media were subsequently autoclaved and poured after reaching about 50°C.

After inoculation, plates were read after 1 week of incubation: signs of clearing or partial clearing/activity in the agar around growth were assessed visually. Thereafter, plates were flooded with 1% CaCl_2_ to precipitate macromolecular mucin. It is reported that mucinase-positive strains will after flooding produce clear zones around the growth(1, 2)_._ *Pseudomonas aeruginosa* ATCC 15692 previously reported to produce zones of clearing on porcine stomach mucin-agar(3) was used as a positive control.

2.5 Growth deferred assay:

Well-isolated colonies of the indicator strain were suspended in sterile 0.85% NaCl and the culture was adjusted to a density corresponding to a McFarland 0.5 standard adjudged spectrophotometrically prior to spraying. The suspension was subsequently diluted 1/20 – 1/150 × in 0.85% NaCl depending on the growth characteristics of the indicator strain. A dilution giving semi-confluent growth was considered optimal. For inoculation, a sterile vaporizer bottle was used with the nozzle held about 20 cm above the plate surface. Spraying of the culture onto the agar plates was performed in a level 2 safety cabinet, and as an additional measure the researcher wore a filter mask and face shield.

2.6 Accession numbers used to create phylogenetic trees

Genomes used to create phylogenetic trees are listed below.

Supplementary Table 1: Accession numbers of the isolates included in the 16S rDNA gene and genome phylogenetic trees for *C.* *macginleyi, Corynebacterium* sp. PCR 32, and the lipase tree.

| **Species name** | **Deposit** | **Biosample accession** |
| --- | --- | --- |
| Corynebacterium kefirresidentii | SB | SAMN06718488 |
| Corynebacterium segmentosum | NCTC 934 | SAMEA37377418 |
| Corynebacterium accolens | ATCC 49725 | SAMN00002226 |
| Corynebacterium aurimucosum | strain DSM 44532 | SAMN16357278 |
| Corynebacterium curieae | c8Ua_181 | SAMN25350282 |
| Corynebacterium intestinale | B5-R-101T | SAMN28196613 |
| Corynebacterium macginleyi | CCUG 32361 | SAMN10236834 |
| Corynebacterium marquesiae | c19Ua_121 | SAMN25350285 |
| Corynebacterium minutissimum | ATCC 23348 | SAMN03140311 |
| Corynebacterium striatum | NBRC 15291 | SAMD00169825 |
| Corynebacterium tuberculostearicum | DSM 44922 | SAMN05878002 |
| Corynebacterium yonathiae | c21Ua_68 | SAMN25350286 |
| Corynebacterium qintianiae | MC1420T | SAMN14086271 |
| Corynebacterium kroppenstedtii | DSM 44385 | SAMN02603033 |
| Corynebacterium urinipleomorphum | Marseille-P2799 | SAMEA47264668 |
| Corynebacterium kalidii | LD5P10 | SAMN27270214 |
| Corynebacterium parakroppenstedtii | MC-26 | SAMN18780376 |
| Corynebacterium zhongnanshanii | zg-320 | SAMN15951547 |
| Corynebacterium heidelbergense | DSM 104638T | SAMN08095970 |
| Corynebacterium tuscaniense | CCUG 51321 | SAMN12771122 |
| Corynebacterium mooreparkense | DSM 44702 | SAMN02603088 |
| Corynebacterium variabile | NBRC 15286 | SAMD00097555 |
| 'Corynebacterium provencense' | SN15 | SAMEA3869306 |
| 'Corynebacterium neomassiliense' | Marseille-P3888 | SAMEA5140071 |
| Corynebacterium glyciniphilum | ATCC 21341 | SAMN03081498 |
| Corynebacterium nuruki | S6-4 | SAMN02470217 |
| Corynebacterium pseudokroppenstedtii | MC-17X | SAMN17488020 |
| Corynebacterium accolens | ATCC 49726 | SAMN00139429 |
| Corynebacterium accolens | ATCC 49725 | SAMN00002226 |
| Corynebacterium accolens | KPL1818 | NZ_AXMA00000000 |

Supplementary results

3.1 Antibiotic susceptibility testing

Supplementary Table 2. Overview of antibiotics susceptibility of *Corynebacterium* isolates

Tests were performed at least twice on separate occasions, and both results are provided in the table (If S, I, R is given once in the table this means both rounds of testing produced the same result).

* no intermediate (I) category. The two values for *C. accolens* (PCR 23) and *C. accolens* (PCR 31) were respectively 1-2 mm to each side of breakpoint diameter

| **Strain** | **Identification based on WGS** | **Antibiotic susceptibility: results scored as S,I,R or zone diameters(mm)** | | | | | | | |
| --- | --- | --- | --- | --- | --- | --- | --- | --- | --- |
|  |  | DA2 | TE30 | MXF5 | CIP5 | VA5 | P1U* | LZD10 | RD5 |
| **PCR1** | *C. propinquum* | R | S | S | I | S | S | S | S |
| **PCR 8** | *C. propinquum* | R | S | S | I | S | S | S | S |
| **PCR 11** | *C. propinquum* | R | S | S | I | S | S | S | S |
| **PCR 2** | *C. marquesiae* | R | S | S | I | S | S | S | S |
| **PCR 3** | *C. marquesiae* | R | S | S | I | S | R | S | R |
| **PCR25** | *C. marquesiae* | R | S | S | I | S | R | S | S |
| **PCR26** | *C. marquesiae* | R | S | S | I | S | S | S | S |
| **PCR27** | *C. marquesiae* | R | S | S | I | S | R | S | R |
| **PCR4** | *C. accolens* | R | S | S | I | S | S | S | S |
| **PCR19** | *C. accolens* | R | S | S | I | S | S | S | S |
| **PCR20** | *C. accolens* | R | S | S | I | S | S | S | S |
| **PCR22** | *C. accolens* | S | S | S | I | S | S | S | S |
| **PCR23** | *C. accolens* | S | S | S | I | S | S,R (borderline values) | S | S |
| **PCR31** | *C. accolens* | R | S | S | I | S | S,R (borderline values) | S | S |
| **PCR7** | *C. macginleyi* | S | S | S | I | S | S | S | S |
| **PCR6** | *C. sanguinis* | R | S | S | I | S | R | S | S |
| **PCR14** | *C. mastitidis* | S | S | S | I | S | R | S | S |
| **PCRF** | *C. mastitidis* | S | S | S | I | S | R | S | S |
| **PCR21** | *C. mastitidis* | S | S | S | I | S | R | S | S |
| **PCR32** | *Corynebacterium* sp. | S | S | S | I | S | S | S | S |
| **ATCC**  **10701** | *C. pseudodiphtheriticum* | S | S | S | I | S | S | S | S |
| **PCR37** | *C. bovis* | Zones were large but not easily measured due to weak growth and unclear edges. | | | | | | | |
| **PCR38** | *C. bovis* |  |  |  |  |  |  |  |  |
| **PCRi** | *C. bovis* |  |  |  |  |  |  |  |  |
| **PCR39** | *C. bovis* |  |  |  |  |  |  |  |  |

3.2 Possible connections between rifampicin resistance in *C.* *marquesiae* and the rpob proteins sequence.

Two isolates (PCR3 and 27) were found to be resistant to rifampicin, whereas isolates PCR 2, 25 and 226 were sensitive. Resistance to rifampicin has been shown to be connected to substitutions/mutations in the antibiotic binding site. PCR 3 and 27 differed from the other isolates (PCR 2, 26 and 25) at two positions shown in supplementary Figure 1. By comparison with studies on other species (4), these substitutions probably fall outside the binding site for rifampicin. If they are involved in resistance, more in-depth analyses such as site -directed mutational studies would need to be performed.


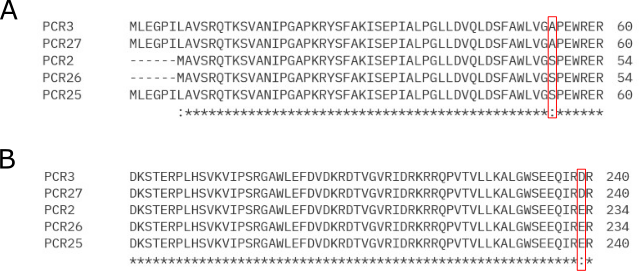


Supplementary Figure 1. Alignment of the rpoB protein sequence extracted from the genomes of *C. marquesiae*. The figure illustrates the 2 positions where the rifampicin resistant isolates differed from the rifampicin sensitive isolates.

Supplementary Table 3 Biochemical summary of *Corynebacterium* isolates. The table summarises the results of biochemical profiling of *Corynebacterium* isolates using the RapID™ CB Plus System for 18 substrates. ND = Not Determined

| **Strain** | **Identification based on WGS** | **Biochemical tests scored +, - or +/-** | | | | | | | | | | | | | | | | | | | |
| --- | --- | --- | --- | --- | --- | --- | --- | --- | --- | --- | --- | --- | --- | --- | --- | --- | --- | --- | --- | --- | --- |
|  |  | GLU | SUC | RIB | MAL | αGLU | βGLU | NAG | GLY1 | ONPG | PHS | EST | PRO | TRY | PYR | LGLY | LEU | URE | NIT | CAT | PIG |
| **ATCC**  **10701** | *C. pseudodiphtheriticum* | - | - | - | - | - | - | - | - | - | + | - | + | + | - | + | + | + | + | + | - |
| **PCR1** | *C. propinquum* | - | - | - | - | - | - | - | - | - | + | - | + | +/- | - | + | + | + | + | + | - |
| **PCR 8** | *C. propinquum* | - | - | - | - | - | - | - | - | - | + | - | + | +/- | - | + | + | + | + | + | - |
| **PCR11** | *C. propinquum* | - | - | - | - | - | - | - | - | - | + | - | + | +/- | - | + | + | + | + | + | - |
| **PCR 2** | *C. marquesiae* | - | - | + | - | - | - | - | - | - | + | - | + | - | +/- | - | - | - | - | + | - |
| **PCR 3** | *C. marquesiae* | + | + | + | - | - | - | - | - | - | + | - | + | - | - | - | +/- | - | - | + | - |
| **PCR25** | *C. marquesiae* | + | + | + | - | - | - | - | - | - | + | - | + | - | - | + | - | - | - | + | - |
| **PCR26** | *C. marquesiae* | + | + | + | - | - | - | - | - | - | + | - | + | - | - | +/- | - | - | - | + | - |
| **PCR27** | *C. marquesiae* | + | + | + | - | - | - | - | - | - | + | - | + | - | - | - | +/- | - | - | + | - |
| **PCR4** | *C. accolens* | + | + | + | - | - | - | - | - | - | +/- | - | + | - | - | + | - | - | + | + | - |
| **PCR19** | *C. accolens* | ND | ND | ND | ND | ND | ND | ND | ND | ND | ND | ND | ND | ND | ND | ND | ND | ND | ND | ND | ND |
| **PCR20** | *C. accolens* | ND | ND | ND | ND | ND | ND | ND | ND | ND | ND | ND | ND | ND | ND | ND | ND | ND | ND | ND | ND |
| **PCR22** | *C. accolens* | +/- | - | +/- | - | - | - | -- | - | - | +/- | +/- | + | - | - | + | - | - | + | + | - |
| **PCR23** | *C. accolens* | + | - | + | - | - | - | - | - | - | +/- | - | + | - | - | + | - | - | +/- | + | - |
| **PCR31** | *C. accolens* | + | + | + | - | - | - | - | - | - | +/- | - | + | - | +/- | + | - | - | + | + | - |
| **PCR7** | *C. macginleyi* | + | + | + | +/- | - | + | - | - | - | + | + | - | - | +/- | - | - | - | + | + | - |
| **PCR6** | *C. sanguinis* | +/- | - | +/- | - | - | - | - | - | - | + | + | + | +/- | - | + | + | + | - | + | - |
| **PCR14** | *C.* *mastitidis* | + | + | + | +/- | + | - | - | - | - | + | + | + | +/- | - | +/- | + | + | - | + | - |
| **PCRF** | *C. mastitidis* | + | + | + | +/- | + | - | - | - | - | + | + | + | +/- | - | +/- | + | + | - | + | - |
| **PCR21** | *C. mastitidis* | + | + | + | +/- | + | - | - | - | - | + | + | +/- | +/- | - | + | + | + | - | + | - |
| **PCR32** | *Corynebacterium* sp. | +/- | - | +/- | +/- | - | - | - | - | - | + | + | + | - | - | +/- | +/- | + | - | + | - |
| **PCR37** | *C. bovis* | +/- | - | +/- | - | + | + | - | - | - | + | + | + | + | + | + | + | + | (+) | + | - |
| **PCR38** | *C. bovis* | + | - | + | - | - | - | - | - | - | + | + | - | + | - | + | + | + | - | + | - |
| **PCRi** | *C. bovis* | + | +/- | + | - | - | - | - | - | - | + | + | + | + | +/- | + | + | + | (+) | + | - |
| **PCR39** | *C. bovis* | + | + | +/- | - | + | + | - | - | - | + | + | +/- | +/- | +/- | +/- | +/- | +/- | - | + | - |

Table 4. Summary of results for deferred growth assay. The table indicates the inhibitory effect of the test strain (spotted centrally on the agar plate) when overlayed with a spray inoculum of the indicator strain. The symbol (+) indicates a zone of inhibition, partial or complete. (+/-) indicates some thinning of the indicator growth around the test strain. (-) indicates no effect. () indicates, not tested. * Ocular isolates obtained from our previous study (5)

| **Test strain**  **Indicator**  **Strain** | ATCC  10701 | PCR1 | PCR8 | PCR11 | PCR2 | PCR3 | PCR25 | PCR26 | PCR27 | PCR4 | PCR19 | PCR20 | PCR22 | PCR23 | PCR31 | PCR7 | PCR6 | PCR14 | PCR21 | PCRF | PCR32 | PCR37 | PCR38 | PCRi | Comments |
| --- | --- | --- | --- | --- | --- | --- | --- | --- | --- | --- | --- | --- | --- | --- | --- | --- | --- | --- | --- | --- | --- | --- | --- | --- | --- |
|  |  | *C. propinquum* | | | *C. marquesiae* | | | | | *C. accolens* | | | | | | *C.*  *macginleyi* | *C. sanguinis* | *C. mastitidis* | | | *Corynebacterium* sp. | *C. bovis* | | |  |
| ***P. aeruginosa***  **DSM22644** | - | - | - | - | - | - | - | - | - | - | - | - | - | - | +/- | - | - | - |  | ¨- | +/- | - | - | - |  |
| ***C. bovis***  **(PCR37)** | - | + | + | + | - | - | - | - | - | - | - | - | - | - | - | + | +/- | + | + | + | + | - | - | - | PCR: 1,8,11 (completely clear zone, 24h) |
| ***S. aureus***  **DSM2569** | - | - | - | - | - | + | - | - | - | - | - | - | - | - | - | +/- | - | + | + | + | + | - | - | - |  |
| ***S. aureus**** | () | () | () | () | () | () | () | () | () | () | () | () | () | () | () | () | () | () | () | () | + | () | () | () |  |
| ***E. faecalis**** | - | () | () | () | - | - | - | () | - | () | () | +/- | () | () | +/- | () | () | () | () | () | + | () | () | () | Figure 1 |
| ***E. faecalis**** | () | () | () | () | () | () | () | () | () | () | () | () | () | () | () | () | () | () | () | () | +/- | () | () | () |  |
| ***C. accolens***  **(PCR20)** | - | () | () | () | () | () | () | () | () | () | () | - | () | () | - | () | () | () | () | () | + | () | () | () | Almost clear zone |
| ***C.***  ***sanguinis***  **(PCR6)** | - | () | () | () | () | () | () | () | () | () | () | + | () | () | + | () | () | () | () | () | + | () | () | () | Figure 1  Almost clear zones |
| ***C. mastitidis***  **PCR21** | - | () | () | () | () | () | () | () | () | () | () | - | () | () | - | () | () | () | () | () | + | () | () | () | Almost clear zone |
| ***C. propinquum***  **PCR11** | - | () | () | () | () | () | () | - | () | () | () | - | () | - | () | () | () | () | () | () | + | - | () | () | Almost clear zone |

.


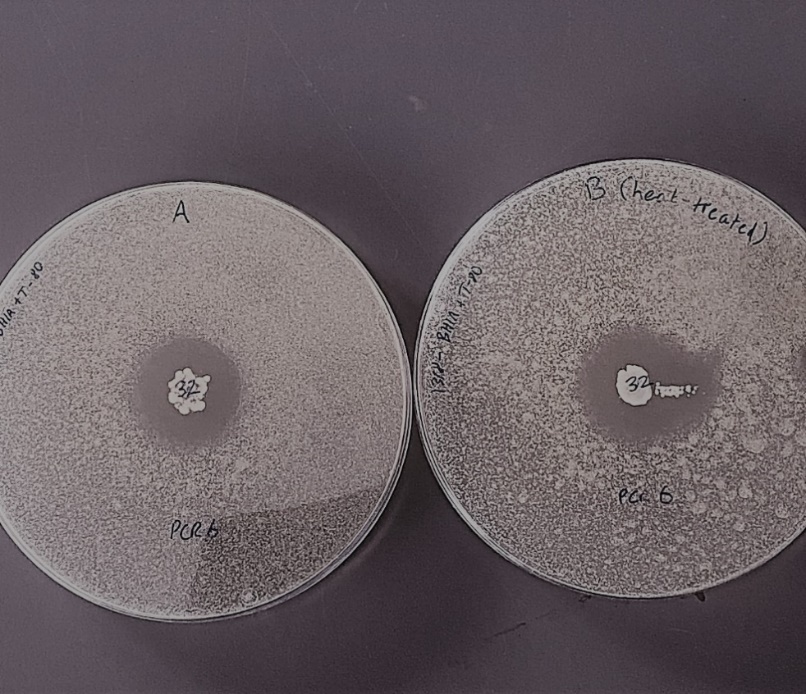


Supplemental Figure 2. *Corynebacterium* sp. PCR 32 (isolated from patient with severe dry eye). Plate A (left of figure). A zone of growth inhibition in a lawn of *Corynebacterium sanguinis* PCR 6 (isolated from patient with mild DE; sprayed onto the plate) is seen round 96h growth of *Corynebacterium* sp. (PCR 32)*.* Plate B (right of figure). Prior to spray-inoculation, the plate containing *Corynebacterium* sp. PCR 32 was treated at 65°C for 1h. Regardless of heat treatment, zones of inhibition of equal sizes were obtained.


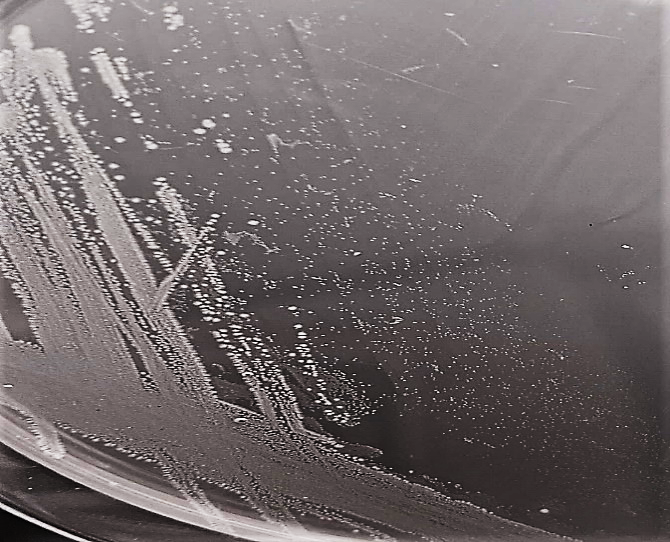

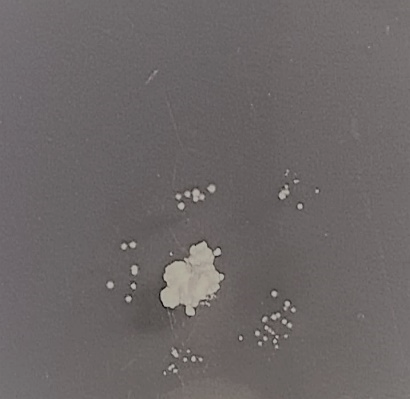


**A B**

Supplemental Figure 3. ‘Goldilocks growth’ of *Corynebacterium* sp. (PCR 32)*.* When inoculated onto an agar plate where it itself is already growing, new colonies only form at a certain distance (~5mm) from the original growth. Panel A: small colonies formed after spraying a suitably diluted bacterial suspension onto the established growth. Note that that the new growth is neither close to not extends far from the original culture. Panel B: here 1 µl aliquots of fresh *Corynebacterium* sp. PCR 32 (about 10 cells) were pipetted in concentric rings at increasing distances from the edge of the established growth out towards the edge of the agar plate. Note that as in A, colonies do not develop close to or far from the primary growth.

Supplemental Table 5. The sequence statistics for the whole genome sequencing of the *Corynebacterium* isolates.

Accession numbers for the isolates are SAMN39854109 - SAMN39854131

| Isolate | Total Reads | Mean Read Length (bp) | Contigs | Total Length (Mbp) | GC (%) | N50 | L50 | Coverage | CDS |
| --- | --- | --- | --- | --- | --- | --- | --- | --- | --- |
| PCR1 | 8,465,634 | 149 | 24 | 2.46 | 56.41 | 296,368 | 3 | 138 | 2,298 |
| PCR 8 | 11,187,260 | 148 | 24 | 2.46 | 56.41 | 302,755 | 3 | 137 | 2,296 |
| PCR11 | 12,838,972 | 148 | 25 | 2.46 | 56.41 | 296,369 | 3 | 138 | 2,301 |
| PCR 2 | 17,690,480 | 148 | 68 | 2.27 | 59.65 | 77,645 | 10 | 143 | 2,176 |
| PCR 3 | 8,176,028 | 147 | 60 | 2.25 | 59.71 | 70,308 | 9 | 143 | 2,166 |
| PCR25 | 16,846,116 | 149 | 61 | 2.27 | 59.66 | 77,645 | 10 | 136 | 2,175 |
| PCR26 | 8,724,140 | 147 | 62 | 2.27 | 59.65 | 77,644 | 10 | 141 | 2,173 |
| PCR27 | 7,482,080 | 148 | 61 | 2.29 | 59.64 | 70,308 | 9 | 140 | 2,227 |
| PCR4 | 7,944,118 | 148 | 39 | 2.52 | 59.44 | 213,344 | 5 | 141 | 2,431 |
| PCR19 | 9,231,024 | 146 | 41 | 2.52 | 59.44 | 213,344 | 5 | 143 | 2,430 |
| PCR20 | 12,737,796 | 149 | 41 | 2.52 | 59.44 | 213,344 | 5 | 139 | 2,430 |
| PCR22 | 12,339,024 | 149 | 23 | 2.43 | 59.65 | 218,278 | 5 | 140 | 2,338 |
| PCR23 | 17,173,010 | 149 | 25 | 2.43 | 59.65 | 218,276 | 4 | 139 | 2,333 |
| PCR31 | 17,407,590 | 149 | 41 | 2.52 | 59.44 | 213,344 | 5 | 138 | 2,433 |
| PCR7 | 8,754,162 | 148 | 61 | 2.39 | 57.18 | 101,411 | 8 | 140 | 2,486 |
| PCR6 | 12,434,644 | 148 | 52 | 2.27 | 65.41 | 93,840 | 8 | 144 | 2,153 |
| PCR14 | 13,856,180 | 148 | 18 | 2.31 | 68.51 | 243,409 | 4 | 142 | 2,278 |
| PCRF | 18,729,116 | 148 | 20 | 2.31 | 68.52 | 240,536 | 5 | 141 | 2,285 |
| PCR21 | 13,322,056 | 148 | 18 | 2.31 | 68.51 | 243,409 | 4 | 142 | 2,286 |
| PCR32 | 15,172,184 | 149 | 13 | 2.12 | 55.52 | 248,601 | 2 | 141 | 2,044 |
| PCR37 | 9,873,006 | 148 | 12 | 2.65 | 72.78 | 701,294 | 2 | 145 | 2,362 |
| PCR38 | 13,297,050 | 148 | 17 | 2.63 | 73.01 | 345,946 | 3 | 144 | 2,321 |
| PCRi | 10,090,968 | 149 | 11 | 2.65 | 72.79 | 375,654 | 3 | 144 | 2,352 |

Supplementary Table 6: the table lists the antibiotic resistance targets identified by BV-BRC for each isolate.

| Resistance target gene | Isolate | Species |
| --- | --- | --- |
| 1-deoxy-D-xylulose 5-phosphate reductoisomerase (EC 1.1.1.267) | PCR i, 37, 38  PCR14, F, 21  PCR 19, 20, 22, 23, 31, 4  PCR 7  PCR 2, 3, 25, 26, 27  PCR 6 | *C. bovis*  *C. mastiditids*  *C. accolens*  *C. macginleyi*  *C. marquesiae*  *C. sanguinis* |
| 16S rRNA (guanine(527)-N(7))-methyltransferase (EC 2.1.1.170) | PCR i, 37, 38  PCR14, F, 21  PCR 19, 20, 22, 23, 31, 4  PCR 7  PCR 2, 3, 25, 26, 27  PCR 6  PCR 32 | *C. bovis*  *C. mastiditids*  *C. acolens*  *C. macginleyi*  *C. marquesiae*  *C. sanguinis*  *Corynebacterium* sp. |
| 3-hydroxyacyl-CoA dehydrogenase (EC 1.1.1.35), FabG4 | PCR i, 37, 38  PCR14, F, 21  PCR 6  PCR 32 | *C. bovis*  *C. mastiditids*  *C. sanguinis*  *Corynebacterium* sp. |
| Alanine racemase (EC 5.1.1.1) | PCR i, 37, 38  PCR14, F, 21  PCR 19, 20, 22, 23, 31, 4  PCR 7  PCR 2, 3, 25, 26, 27  PCR 6  PCR 32 | *C. bovis*  *C. mastiditids*  *C. accolens*  *C. macginleyi*  *C. marquesiae*  *C. sanguinis*  *Corynebacterium* sp. |
| CDP-diacylglycerol--glycerol-3-phosphate 3-phosphatidyltransferase (EC 2.7.8.5) | PCR i, 37, 38  PCR14, F, 21  PCR 19, 20, 22, 23, 31, 4,  PCR 7  PCR 2, 3, 25, 26, 27  PCR 6  PCR 32 | *C. bovis*  *C. mastiditids*  *C. accolens*  *C. macginleyi*  *C. marquesiae*  *C. sanguinis*  *Corynebacterium* sp. |
| D-alanine--D-alanine ligase (EC 6.3.2.4) | PCR i, 37, 38  PCR14, F, 21  PCR 19, 20, 22, 23, 31, 4  PCR 7  PCR 2, 3, 25, 26, 27  PCR 6  PCR 32 | *C. bovis*  *C. mastiditids*  *C. accolens*  *C. macginleyi*  *C. marquesiae*  *C. sanguinis*  *Corynebacterium* sp. |
| DNA gyrase subunit A (EC 5.99.1.3) | PCR i, 37, 38  PCR14, F, 21  PCR 19, 20, 22, 23, 31, 4  PCR 7  PCR 2, 3, 25, 26, 27  PCR 6  PCR 32 | *C. bovis*  *C. mastiditids*  *C. accolens*  *C. macginleyi*  *C. marquesiae*  *C. sanguinis*  *Corynebacterium* sp. |
| DNA gyrase subunit B (EC 5.99.1.3) | PCR i, 37, 38  PCR14, F, 21  PCR 19, 20, 22, 23, 31, 4  PCR 7  PCR 2, 3, 25, 26, 27  PCR 6  PCR 32 | *C. bovis*  *C. mastiditids*  *C. accolens*  *C. macginleyi*  *C. marquesiae*  *C. sanguinis*  *Corynebacterium* sp. |
| DNA-directed RNA polymerase beta subunit (EC 2.7.7.6) | PCR i, 37, 38  PCR14, F, 21  PCR 19, 20, 22, 23, 31, 4  PCR 7  PCR 6  PCR 32 | *C. bovis*  *C. mastiditids*  *C. accolens*  *C. macginleyi*  *C. sanguinis*  *Corynebacterium* sp. |
| DNA-directed RNA polymerase beta' subunit (EC 2.7.7.6) | PCR i, 37, 38  PCR14, F, 21  PCR 19, 20, 22, 23, 31, 4  PCR 7  PCR 2, 3, 25, 26, 27  PCR 6  PCR 32 | *C. bovis*  *C. mastiditids*  *C. accolens*  *C. macginleyi*  *C. marquesiae*  *C. sanguinis*  *Corynebacterium* sp. |
| Dihydrofolate reductase (EC 1.5.1.3) | PCR i, 37, 38  PCR14, F, 21  PCR 19, 20, 22, 23, 31, 4  PCR 7  PCR 2, 3, 25, 26, 27  PCR 6  PCR 32 | *C. bovis*  *C. mastiditids*  *C. accolens*  *C. macginleyi*  *C. marquesiae*  *C. sanguinis*  *Corynebacterium* sp. |
| Dihydropteroate synthase (EC 2.5.1.15) | PCR i, 37, 38  PCR14, F, 21  PCR 19, 20, 22, 23, 31, 4  PCR 7  PCR 2, 3, 25, 26, 27  PCR 6  PCR 32 | *C. bovis*  *C. mastiditids*  *C. accolens*  *C. macginleyi*  *C. marquesiae*  *C. sanguinis*  *Corynebacterium* sp. |
| Glycerophosphoryl diester phosphodiesterase (EC 3.1.4.46) | PCR i, 37, 38  PCR14, F, 21  PCR 19, 20, 22, 23, 31, 4  PCR 7  PCR 2, 3, 25, 26, 27  PCR 6  PCR 32 | *C. bovis*  *C. mastiditids*  *C. accolens*  *C. macginleyi*  *C. marquesiae*  *C. sanguinis*  *Corynebacterium* sp. |
| Hydrogen peroxide-inducible genes activator => OxyR | PCR i, 37, 38  PCR14, F, 21  PCR 19, 20, 22, 23, 31, 4  PCR 7  PCR 2, 3, 25, 26, 27  PCR 6  PCR 32 | *C. bovis*  *C. mastiditids*  *C. accolens*  *C. macginleyi*  *C. marquesiae*  *C. sanguinis*  *Corynebacterium* sp. |
| Isoleucyl-tRNA synthetase (EC 6.1.1.5) | PCR i, 37, 38  PCR14, F, 21  PCR 19, 20, 22, 23, 31, 4  PCR 7  PCR 2, 3, 25, 26, 27  PCR 6  PCR 32 | *C. bovis*  *C. mastiditids*  *C. accolens*  *C. macginleyi*  *C. marquesiae*  *C. sanguinis*  *Corynebacterium* sp. |
| Probable (3R)-hydroxyacyl-CoA dehydratase HtdX | PCR i, 37, 38  PCR14, F, 21  PCR 32 | *C. bovis*  *C. mastiditids*  *Corynebacterium* sp. |
| SSU ribosomal protein S10p (S20e) | PCR i, 37, 38  PCR14, F, 21  PCR 19, 20, 22, 23, 31, 4  PCR 7  PCR 2, 3, 25, 26, 27  PCR 6  PCR 32 | *C. bovis*  *C. mastiditids*  *C. accolens*  *C. macginleyi*  *C. marquesiae*  *C. sanguinis*  *Corynebacterium* sp. |
| SSU ribosomal protein S12p (S23e) | PCR i, 37, 38  PCR14, F, 21  PCR 19, 20, 22, 23, 31, 4  PCR 7  PCR 2, 3, 25, 26, 27  PCR 6  PCR 32 | *C. bovis*  *C. mastiditids*  *C. accolens*  *C. macginleyi*  *C. marquesiae*  *C. sanguinis*  *Corynebacterium* sp. |
| Transcription termination factor Rho | PCR i, 37, 38  PCR14, F, 21  PCR 19, 20, 22, 23, 31, 4  PCR 7  PCR 2, 3, 25, 26, 27  PCR 6  PCR 32 | *C. bovis*  *C. mastiditids*  *C. accolens*  *C. macginleyi*  *C. marquesiae*  *C. sanguinis*  *Corynebacterium* sp. |
| Translation elongation factor G | PCR i, 37, 38  PCR14, F, 21  PCR 19, 20, 22, 23, 31, 4  PCR 7  PCR 2, 3, 25, 26, 27  PCR 6  PCR 32 | *C. bovis*  *C. mastiditids*  *C. accolens*  *C. macginleyi*  *C. marquesiae*  *C. sanguinis*  *Corynebacterium* sp. |
| Translation elongation factor Tu | PCR i, 37, 38  PCR14, F, 21  PCR 19, 20, 22, 23, 31, 4  PCR 7  PCR 2, 3, 25, 26, 27  PCR 6  PCR 32 | *C. bovis*  *C. mastiditids*  *C. accolens*  *C. macginleyi*  *C. marquesiae*  *C. sanguinis*  *Corynebacterium* sp. |
| Two component system response regulator MtrA | PCR i, 37, 38  PCR14, F, 21  PCR 19, 20, 22, 23, 31, 4  PCR 7  PCR 2, 3, 25, 26, 27  PCR 6  PCR 32 | *C. bovis*  *C. mastiditids*  *C. accolens*  *C. macginleyi*  *C. marquesiae*  *C. sanguinis*  *Corynebacterium* sp. |
| Two component system sensor histidine kinase MtrB | PCR i, 37, 38  PCR14, F, 21  PCR 19, 20, 22, 23, 31, 4  PCR 7  PCR 2, 3, 25, 26, 27  PCR 6  PCR 32 | *C. bovis*  *C. mastiditids*  *C. accolens*  *C. macginleyi*  *C. marquesiae*  *C. sanguinis*  *Corynebacterium* sp. |
| UDP-N-acetylglucosamine 1-carboxyvinyltransferase (EC 2.5.1.7) | PCR i, 37, 38  PCR14, F, 21  PCR 19, 20, 22, 23, 31, 4  PCR 7  PCR 2, 3, 25, 26, 27  PCR 6  PCR 32 | *C. bovis*  *C. mastiditids*  *C. accolens*  *C. macginleyi*  *C. marquesiae*  *C. sanguinis*  *Corynebacterium* sp. |
| 23S rRNA (adenine(2058)-N(6))-dimethyltransferase (EC 2.1.1.184) => Erm(X) | PCR 19, 20, 31, 4 | *C. accolens* |
| 23S rRNA (adenine(2058)-N(6))-dimethyltransferase (EC 2.1.1.184) => Erm38/39/40 | PCR 6 | *C. sanguinis* |

Pangenome analysis

The pangenome analysis was performed using the BPGA pipeline and showed an open pangenome (Supplementary Figure 4).


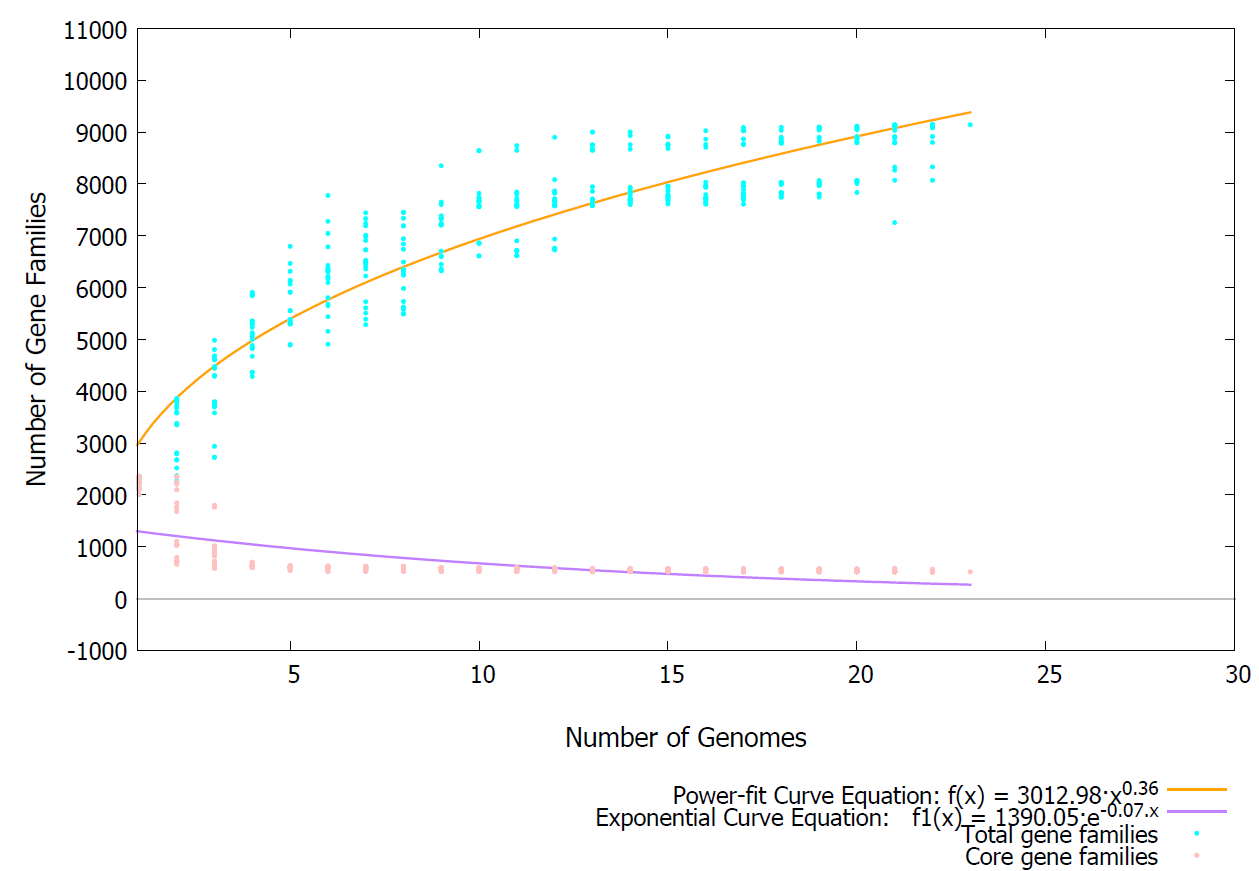


Supplemental Figure 4. Pan (orange line) and core (purple line) genome profile of isolates included in this study.

Supplementary discussion on lipolytic activity (and other candidate antimicrobial factors) in ocular *Corynebacterium* isolates in this study:

*C. accolens*

All *C. accolens* isolates tested negative for esterase activity which is the expected kit result (0% positive). However, perusal of the some 30 culture collection strains of *C. accolens* registered in BacDrive (<https://bacdive.dsmz.de/strain/149844>) shows, albeit based on other kits, that C-4 and C-8 esterase/lipase activities are not uncommon in the species. The annotated *C. accolens* (PCR 20 and PCR 22) and *C. macginleyi* (PCR 7) genomes contained sequences that were highly similar to TAG secreted lipases, a finding which is in line with the strong lipolytic activities seen on olive oil agar (Table 1). These sequences clustered (Figure 8)with the TAG lipase (StrainKPL1818-WP034657805.1-*Corynebacterium*-sp) shown by Bomar et al., (6) to cleave the TAG triolein releasing oleic acid which inhibited growth of *Streptococcus pneumoniae*. Also found in this clade (coloured blue in Figure 8) is an enzyme sequence from the *C. macginleyi* isolate (PCR 7) which showed weak hydrolysis of olive oil. Most sequences making up the blue clade in Figure 8 contained a standard secretory signal sequence peptide, and the canonical motif G X S X G which is found at the active site of many lipases. However, although a signal peptide was predicted for the *C. accolens* GenBank sequences and the *C. macginleyi* isolate enzyme, there were only very weak indications of a secretory signal peptide for the predicted *C. accolens* enzymes (1a and 2a) from isolates PCR 20 and PCR 22. If the lipase activity demonstrated in the olive oil test and the results of the deferred growth inhibition assay for the *C. accolens* isolates are the result of the activity of these enzymes, then they likely must be released from the cell by mechanisms that are not dependent on the general secretion pathway.

*Corynebacterium* sp. PCR 32

The lipase sequences present in the red clade in Figure 8 represent GenBank sequences obtained mainly from the type strains of *C. kroppenstedtii*, *C. parakroppenstedtii* and *C. pseudokroppenstedtii.* A lipase from *Corynebacterium* sp. isolate (PCR 32) aligned in this clade group. Members of the clade group lacked the canonical GxGxS sequence of ‘true’ TAG lipases but a somewhat similar GNSAR was present in all. Searches on the annotated *Corynebacterium* sp. isolate (PCR 32) genome produced no sequences with significant sequence identity of the secreted TAG lipase type present in *C. accolens*; a finding supported by the weak activity seen on olive oil agar (Table 1). A second lipase sequence determinant, *Corynebacterium* sp. PCR 32(a), showed only low sequence identity (<40%) with other sequences. It contained the canonical G X S X G motif, but not a signal peptide. Nothwithsatnding, both sequences were predicted to be TAG lipases with a high degree of certainty using the PhiGnet function-prediction tool (7) which harnesses evolutionary data extracted from multiple sequence alignments.

*C. bovis*

The *C. bovis* isolate (PCR37) did not produce lipase activity on olive oil agar (table 1), but esterase activity was found with the RapID™ CB Plus System (table 4). Three putative secreted lipases [*C. bovis* (b), (c), (d) Figure 8] contain both the sequence motif GXSXG and a classical secretory signal peptide. In both instances, InterPro returns sequence similarity with both the secreted lipases family ([IPR005152](https://www.ebi.ac.uk/interpro/entry/InterPro/IPR005152/)) and the esterase LipA family (PIRSF029) over almost the entire sequence length. If the substrate specificity for these enzymes is most akin to esterases, this would be in line with the observed results of the olive oil agar (negative) and kit esterase (positive) tests. A fourth sequence *C. bovis* (a) has the canonical motif, but no indications of a secretory signal peptide. Interpro shows some regions of sequence similarity with the [Lipase class - 2 (PF01674](https://www.ebi.ac.uk/interpro/entry/pfam/PF01674/)) family.

Szabo et al (8) performed whole genome sequencing on 22 nasopharyngeal isolates representing 4  *Corynebacterium* species (not including *C. macginleyi*) , and only found TAG lipases of the sequence family forming the blue clade in Figure 8 in the *C. accolens* isolates, which is in accord with the results of the present study.

Growth deferred assay and possible connections to isolate lipolytic activity

All the isolates were tested for antibacterial activity against a 3-strain panel of *P. aeruginosa, S. aureus and C. bovis*. In addition, some isolates were tested for growth inhibition of additional indicator bacteria (Supplementary Table 4). None of the *C. accolens* isolates showed clear inhibition of *S. aureus* and *C. bovis*. However, PCR 31 showed very weak inhibition of *P. aeruginosa* (Supplementary Table 4). *C. accolens* PCR20 the source of putative lipase *C. accolens* a(a)(Figure 8) was tested against a wider panel of indicator strains and was seen to clearly inhibit the growth of *Enterococcus* *faecalis* and *C. sanguinis* (PCR 6). The *C.* *macginleyi* isolate (PCR 7) which was the source of lipase *C*. *macginleyi* (a)(Figure 8) showed inhibition of *C. bovis* and some activity against *S. aureus* (Supplementary Table 4). The results confirm the findings of others that a number of *Corynebacterium* species can inhibit the growth of pathogens, including pertinently *S. aureus* which is a common ocular pathogen, - but our study seems to be the first report of intra-genus activity against other *Corynebacterium* species. Bomar et al., (2016) (6) found that *C. accolens* KPL1818 extracellular TAG lipase activity (KPL1818 (a) in Figure 8) produces oleic acid from triolein and that this inhibits *Streptococcus pneumoniae.* The culture medium used in our deferred growth inhibition assay contained 0.8% Tween 80 (polyoxyethylenesorbitan mono-oleate), a synthetic lipid that contains an ester of oleic acid, a monounsaturated 18-carbon fatty acid. *C. kroppenstedtii* (9), *C. accolens* (6) and *C. bovis* have lipid-requiring lifestyles and grow poorly on BHIA unless supplemented with Tw80. Strains of these species are thus presumably able to produce-lipolytic enzymes which hydrolyze the ester bond of Tw80 releasing oleic acid and other fatty acids to support growth. Even though Figure 8 in the present work shows these must be structurally different classes. The source of Tw80 used in our study contains mainly Oleic acid (≥58.0%) with a balance primarily linoleic, palmitic, and stearic acids. Presumably secreted lipases constituting the blue coloured clade in Figure 8, can free oleic acid from Tw80 in the agar, and this could account for the results of the deferred growth inhibition assay. However, Bomar et al.,(6) also reported that a secreted TAG-lipase deficient mutant grew comparably to the wild type in medium supplemented with Tw80, suggesting that still other lipolytic enzymes are able to hydrolyse Tw80. Plou et al., (10) evaluated Tw80 as an esterase/ lipase substrate for lipolytic activity, and concluded that Tw80 occupies an intermediate position in the map of esterase/ lipase substrates, and that it is not a simple esterase substrate as had been suggested due to its solubility in water. Thus, if hydrolysis of Tw80 producing growth inhibiting concentrations of fatty acids is the major reason for the results of the deferred growth assay, other lipases shown in Figure 8 lying outside of the blue clade, and perhaps also esterases, could explain the growth inhibitory effect seen with particularly the *Corynebacterium* sp. 32 isolate which lacks *typical* genetic determinants of secretory lipases.

In most instances, the strongest inhibitory effects (largest and clearest zones) were seen when using the *Corynebacterium* sp. 32 isolate as antagonist (see supplementary Table 4; Figure 1 and supplementary Figure 2). The isolate inhibited the growth of all the extended panel indicator strains, although the effect against *P. aeruginosa* was very weak. The strain produced esterase (Supplemental Table 3) and was weakly positive for lipase on olive oil agar. Thus, lipolytic agents are leaving the cell and could be hydrolyzing Tw80 producing antimicrobial fatty acids. What we considered the best candidates for this activity are shown in Figure 8 and discussed above and in main body of this report. To see if the inhibitory agent(s) were heat stable, agar plates with *Corynebacterium* sp. PCR32 growth were warmed to 65°C for 1h before spraying on the indicator, *C. sanguinis*. The resulting zones of inhibition (with and without heating) were exactly similar in size and appearance (supplemental Figure 2), indicating that the agent(s) were heat stable. This would be the expected result if the inhibitory substance(s) was fatty acids formed from Tw80 in the agar prior to heat treatment – ie during growth of the strain prior to spraying of the indicator organisms (see materials and methods). However, the case for growth inhibition activities of *Corynebacterium* sp. PCR32 relating to extracellular lipase activity is not as well supported by the genomic data on lipolytic enzymes as it is for *C. accolens* as discussed above. Another possible candidate for inhibitory activity is the presence of heat-stable bacteriocins. It has previously been shown that certain *Corynebacterium* species produce various bacteriocins, bacteriocin-like substances and biosurfactants (11) which inhibit the growth of opportunistic microorganisms and their biofilm formation (12, 13, 14, 15, 16, 17). Gross and Vidaver, (17) demonstrated that the majority (85% of all strains tested) of 12 phytopathogenic *Corynebacterium* species produced bacteriocin(s) on nutrient broth- yeast extract (NBY) medium. Most of the bacteriocins appeared to be low molecular weight and diffusible proteins resistant to heat, but sensitive to proteolytic enzymes including proteinase K. The annotated genome of *Corynebacterium* sp. PCR32 was searched for biosynthetic loci potentially involved in the production of bacteriocins using the Antismash® (<https://antismash.secondarymetabolites.org/#!/start>) pipeline, and for bacteriocins by manual search of all predicted proteins between 80 and 300 residues in the annotated genome (Motif Search, BLASTP and FASTA). In addition, sequences deposited as *Corynebacterium* bacteriocins in GenBank were compared with the annotated genome. The most promising predicted peptide contained a 26-residue secretory signal sequence. After removal of the signal sequence, the resultant 96 residue peptide was analysed using motif search but showed only very low sequence identity (Position 36-78; Independent E-value, 0.02) to a class II bacteriocin with a double-glycine leader peptide (PF10439). Thus, the inhibitory effects of *Corynebacterium* sp. PCR32 seen in the present study if not related to lipolytic activity were not readily explicable. It is possible that some bacterial products may act as surfactants affecting in a more general manner the growth of other species, but this remains a hypothesis.

The *Corynebacterium* sp. PCR32 isolate showed an unusual and unique growth response (Supplemental Figure 3). Repeated attempts to use the isolate as an indicator strain consistently resulted in scant or no growth on the plate onto which it was sprayed. On inoculation in low numbers/density onto an agar plate where it was already growing (as in the growth deferred assay approach), we serendipitously noticed what we have called a goldilocks effect - where the inoculum would neither grow close to nor at a distance from itself (see supplementary Figure 3A and 3B for alternative demonstrations of this effect). The finding would fit a model where both growth-stimulatory and growth-inhibitory substances were being produced from pre-established high-density growth, such that only at certain distance from the central patch of thick growth is the concentration of growth stimulatory agent(s) sufficiently high and the concentration of growth-inhibitory substance(s) sufficiently low to allow growth of dilute inoculums. The potential advantages, if any, gained by this response are not easily understood, and it might be a situation that only has relevance under laboratory culture on agar.

The annotated *Corynebacterium* sp. PCR32 genome shows the presence of a putative neocarzinostatin apoprotein domain-containing protein. Neocarzinostatin (NCS) is a member of the macromolecular antitumour antibiotic family. NCS consists of two components, a polypeptide (apo-NCS) and a nonprotein chromophore. Neocarzinostatin was first isolated in culture supernatants of *Streptomyces carzinostaticus* (18) and has both antitumor and antibacterial effects; targeting DNA. Koide et al.,(19) demonstrated good antibacterial activity against a range of Gram-positive bacteria. It is thus possible that *Corynebacterium* sp. PCR32 is able to produce NCS or an NCS-like molecule and that this could contribute to its demonstrated antibacterial effects.

1. Gessner AR, Mortensen JE. Pathogenic factors of Pseudomonas cepacia isolates from patients with cystic fibrosis. Journal of Medical Microbiology. 1990;33(2):115-20.

2. van der Wiel-Korstanje JAA, Winkler KC. The Faecal Flora In Ulcerative Colitis. Journal of Medical Microbiology. 1975;8(4):491-501.

3. Jacob KM, Reguera G. Competitive advantage of oral streptococci for colonization of the middle ear mucosa. Biofilm. 2022;4:100067.

4. Cai X-C, Xi H, Liang L, Liu J-D, Liu C-H, Xue Y-R, et al. Rifampicin-Resistance Mutations in the rpoB Gene in Bacillus velezensis CC09 have Pleiotropic Effects. Frontiers in Microbiology. 2017;8.

5. Naqvi M, Fineide F, Utheim TP, Charnock C. Culture- and non-culture-based approaches reveal unique features of the ocular microbiome in dry eye patients. The Ocular Surface. 2024.

6. Bomar L, Brugger SD, Yost BH, Davies SS, Lemon KP. Corynebacterium accolens Releases Antipneumococcal Free Fatty Acids from Human Nostril and Skin Surface Triacylglycerols. mBio. 2016;7(1):10.1128/mbio.01725-15.

7. Jang YJ, Qin Q-Q, Huang S-Y, Peter ATJ, Ding X-M, Kornmann B. Accurate prediction of protein function using statistics-informed graph networks. Nature Communications. 2024;15(1):6601.

8. Szabo D, Ostorhazi E, Stercz B, Makra N, Penzes K, Kristof K, et al. Specific nasopharyngeal Corynebacterium strains serve as gatekeepers against SARS-CoV-2 infection. GeroScience. 2023;45(5):2927-38.

9. Tauch A, Fernández-Natal I, Soriano F. A microbiological and clinical review on Corynebacterium kroppenstedtii. International Journal of Infectious Diseases. 2016;48:33-9.

10. Plou FJ, Ferrer M, Nuero OM, Calvo MV, Alcalde M, Reyes F, et al. Analysis of Tween 80 as an esterase/ lipase substrate for lipolytic activity assay. Biotechnology Techniques. 1998;12(3):183-6.

11. Dwivedi A, Kumar A, Bhat JL. Production and Characterization of Biosurfactant from Corynebacterium Species and Its Effect on the Growth of Petroleum Degrading Bacteria. Microbiology. 2019;88(1):87-93.

12. Efthimia P, Sebastian JR, Dominik W, Bernhard JE, Christian UR, Oliver G. Identification, Characterization and Mode of Action of Corynaridin, a Novel Linaridin from &lt;em&gt;Corynebacterium lactis&lt;/em&gt. bioRxiv. 2022:2022.05.11.491181.

13. Kwaszewska A, Szewczyk EM. [Production of antibacterial substances by resident corynebacteria isolated from human skin]. Med Dosw Mikrobiol. 2007;59(3):251-7.

14. Gladysheva IV, Khlopko YA, Cherkasov SV, Kataev VY. Genome sequence of Corynebacterium amycolatum ICIS 99 isolated from human vagina reveals safety and beneficial properties. Archives of Microbiology. 2022;204(4):226.

15. Gladysheva IV, Cherkasov SV. Antibiofilm activity of cell-free supernatants of vaginal isolates of Corynebacterium amycolatum against Pseudomonas aeruginosa and Klebsiella pneumoniae. Archives of Microbiology. 2023;205(4):158.

16. Wysocki P, Kwaszewska AK, Szewczyk EM. [Influence of substances produced by lipophilic Corynebacterium CDC G1 ZMF 3P13 on the microorganisms inhabiting human skin]. Medycyna doswiadczalna i mikrobiologia. 2011;63(1):45-52.

17. Gross DC, Vidaver AK. Bacteriocins of phytopathogenic Corynebacterium species. Can J Microbiol. 1979;25(3):367-74.

18. Ishida N, Miyazaki K, Kumagai K, Rikimaru M. NEOCARZINOSTATIN, AN ANTITUMOR ANTIBIOTIC OF HIGH MOLECULAR WEIGHT. ISOLATION, PHYSIOCHEMICAL PROPERTIES AND BIOLOGICAL ACTIVITIES. J Antibiot (Tokyo). 1965;18:68-76.

19. Koide Y, Ishii F, Hasuda K, Koyama Y, Edo K, Kitamine S, et al. Isolation of a non-protein component and a protein component from neocarzinostatin (NCS) and their biological activities. J Antibiot (Tokyo). 1980;33(3):342-6.
